# Distribution of insecticide resistance and mechanisms involved in the arbovirus vector *Aedes aegypti* in Laos and implication for vector control

**DOI:** 10.1371/journal.pntd.0007852

**Published:** 2019-12-12

**Authors:** Sébastien Marcombe, Bénédicte Fustec, Julien Cattel, Somesanith Chonephetsarath, Phoutmany Thammavong, Nothasin Phommavanh, Jean-Philippe David, Vincent Corbel, Ian W. Sutherland, Jeffrey C. Hertz, Paul T. Brey

**Affiliations:** 1 Medical Entomology and Vector-Borne Disease Laboratory, Institut Pasteur du Laos, Vientiane, Laos; 2 Maladies Infectieuses et Vecteurs, Ecologie, Génétique, Evolution et Contrôle, Institut de Recherche pour le Développement (IRD), Université de Montpellier, Montpellier, France; 3 Laboratoire d'Ecologie Alpine (LECA), UMR 5553 CNRS - Université Grenoble-Alpes, Grenoble, France; 4 U.S. Naval Medical Research Unit TWO, Singapore; University of California, Davis, UNITED STATES

## Abstract

**Background:**

The yellow fever mosquito *Aedes aegypti* is the major vector of dengue, yellow fever, Zika, and Chikungunya viruses. Worldwide vector control is largely based on insecticide treatments but, unfortunately, vector control programs are facing operational challenges due to mosquitoes becoming resistant to commonly used insecticides. In Southeast Asia, resistance of *Ae*. *aegypti* to chemical insecticides has been documented in several countries but no data regarding insecticide resistance has been reported in Laos. To fill this gap, we assessed the insecticide resistance of 11 *Ae*. *aegypti* populations to larvicides and adulticides used in public health operations in the country. We also investigated the underlying molecular mechanisms associated with resistance, including target site mutations and detoxification enzymes putatively involved in metabolic resistance.

**Methods and results:**

Bioassays on adults and larvae collected in five provinces revealed various levels of resistance to organophosphates (malathion and temephos), organochlorine (DDT) and pyrethroids (permethrin and deltamethrin). Synergist bioassays showed a significant increased susceptibility of mosquitoes to insecticides after exposure to detoxification enzyme inhibitors. Biochemical assays confirmed these results by showing significant elevated activities of cytochrome P450 monooxygenases (P450), glutathione S-transferases (GST) and carboxylesterases (CCE) in adults. Two *kdr* mutations, V1016G and F1534C, were detected by qPCR at low and high frequency, respectively, in all populations tested. A significant negative association between the two *kdr* mutations was detected. No significant association between *kdr* mutations frequency (for both 1534C and 1016G) and survival rate to DDT or permethrin (*P* > 0.05) was detected. Gene Copy Number Variations (CNV) were detected for particular detoxification enzymes. At the population level, the presence of CNV affecting the carboxylesterase *CCEAE3A* and the two cytochrome P450 *CYP6BB2* and *CYP6P12* were significantly correlated to insecticide resistance.

**Conclusions:**

These results suggest that both *kdr* mutations and metabolic resistance mechanisms are present in Laos but their impact on phenotypic resistance may differ in proportion at the population or individual level. Molecular analyses suggest that CNV affecting *CCEAE3A* previously associated with temephos resistance is also associated with malathion resistance while CNV affecting *CYP6BB2* and *CYP6P12* are associated with pyrethroid and possibly DDT resistance. The presence of high levels of insecticide resistance in the main arbovirus vector in Laos is worrying and may have important implications for dengue vector control in the country.

## Author summary

*Aedes aegypti* is the major vector of dengue in Laos and the control of this vector rely mainly on insecticide treatments. Compared to the neighboring countries, where resistance has been detected, there was no data on the distribution, the levels, and the mechanisms involved in the resistance in Laos. Laboratory bioassays showed that resistance to the currently used larvicides (temephos) and adulticides (pyrethroids) was present at different levels and distributed throughout the country. This may have important implications for dengue vector control in Laos. The mechanisms underlying the resistance were determined to be both metabolic and target site mutations (*kdr*) supporting results found in other countries. Several key detoxification enzyme genes were identified as potential candidates for metabolic resistance. This study provides a baseline on insecticide resistance in Laos and will help the Public Health authorities in designing more adapted vector control strategies.

## Introduction

Because of changes in the environment, climate, and frequency of transportation during the last decades, the world has seen a dramatic resurgence of emerging and reemerging arboviral diseases such as dengue, Zika and chikungunya fever. The *Aedes aegypti* mosquito is the main vector of these important diseases and according to the World Health Organization (WHO), 2.5 billion people live in an area at risk of transmission of one or more arboviruses [1]. In Laos, dengue is reemerging and there have been outbreaks of all four serotypes in the country, both in rural and urban areas [2–7]. The most recent important dengue outbreak was in 2013 with 44,098 cases and 95 deaths reported [2,7]. Between 2014 and 2017, the number of annually reported cases varied from 2,000 to 18,000 with 10 fatalities per year [7]. Even if the presence of CHIKV was suspected before [6,8], the first authenticated cases of active chikungunya virus infection involving *Ae*. *aegypti* was detected during the 2012–2013 outbreak in Southern Laos [9,10]. The increasing incidence of chikungunya and dengue in Laos, and Southeast Asia (SEA), is deleterious to the health, livelihood, and economy throughout the country [11]. Autochthonous transmission of ZIKV has not been detected in Laos but this specific disease is not particularly targeted by the Lao public health authorities.

Because of the absence of effective vaccines or specific treatment against these diseases, vector control remains the only strategy for reducing the transmission and preventing outbreaks. Public health vector control strategies rely on active community participation, health education programs, and environmental management that include improvement of water supplies and storage, solid waste management, and modification of human-made larval habitats worldwide [1,12]. In Laos, these strategies are at an early stage of development despite the organized efforts to promote community participation in the dengue control program since the 1990’s [13,14]. Chemical control methods using insecticides against larvae and adult mosquitoes have been routinely conducted by public health services for decades. The organochlorine (OC) DDT has been used in the country for vector control and agriculture from the 1950’s until it was banned in 1989 [15]. Then, the Centers for Disease Control and Prevention (CDC) in Laos relied on the use of the larvicide temephos (organophosphate [OP] family, Abate formulation) that was first used during the dengue outbreak of 1987 [14]. This insecticide formulation is used to treat large water containers and is distributed throughout the country as an active measure to prevent mosquitoes from developing in known breeding sites that cannot be removed or protected; it is also applied upon in areas where dengue cases are reported. The OP malathion has been used in the country since the 1990’s for thermal fogging applications to reduce adult mosquito populations and other adulticides such as deltamethrin and permethrin from the pyrethroid family (PYR) have been used since the early 2000’s. The use of these insecticides for decades may have induced selective pressures that may reduce the efficacy of the current vector control operations, resistance alleles have potentially been circulating in Laos as described in neighboring countries [16,17]. Resistance of *Ae*. *aegypti* to the OC dieldrin and DDT were first detected in SEA in the 1960s [18]. During the same period, first evidence of malathion and temephos resistance was detected in Thailand, Cambodia and Myanmar [18]. More recently, strong levels of resistance to OPs and PYRs have been reported in *Ae*. *aegypti* populations in SEA including the neighboring countries of Laos (i.e. Thailand, China, Vietnam and Cambodia;[16,19–23].

Insecticide resistance in *Ae*. *aegypti* is mainly associated with the over-expression of detoxification enzymes (metabolic-based resistance) and/or mutations in the sequence of the target protein that induce insensitivity to the insecticide (target-site resistance). The main target site resistance mechanisms known in *Ae*. *aegypti* involve amino acid substitutions in the voltage gated sodium channel (VGSC) that cause a resistance to DDT/pyrethroid insecticides known as knockdown resistance (*kdr*) [24–27]. In *Ae*. *aegypti*, 11 *kdr* mutations at 9 different codon positions in the VGSC domains I-IV have been reported [17,28]. In SEA, three mutations (S989P, V1016G and F1534C) have been reported to confer resistance to PYR [22,29–32]. The V1016G mutation causes insensitivity to permethrin and deltamethrin, while the F1534C mutation confers resistance only to permethrin [29]. The S989P mutation causes no or very little resistance to pyrethroids [29]. However, very high levels of pyrethroid resistance have been reported with individuals carrying two (S989P+V1016G) or three mutations (S989P+V1016G+F1534C) [29,33]. Individuals carrying those three mutations have been reported in India, Myanmar, Saudi Arabia and, Singapore [21,32,34,35].

Metabolic-based resistance involves the bio-transformation and/or sequestration of the insecticide by detoxifying enzymes [17,25]. Three large enzyme families are frequently associated with resistance: the cytochrome P450 monooxygenases (P450s), the glutathione S-transferases (GSTs) and the carboxy/cholinesterases (CCEs) [25,36–39]. In SEA, several studies support the importance of metabolic mechanisms in the resistance of *Ae*. *aegypti* to PYRs and OPs [23,40,41] with the involvement of the three detoxification enzyme families at different levels. As over expression is frequently associated with over transcription, most candidate genes have been identified by comparing gene expression profiles between susceptible and resistant populations. The over expression of multiple P450 genes has been associated with PYR resistance in Singapore populations [32]. In turn, OPs resistance was rather associated with increased CCE activities in Thailand where the genes *CCEAE3A* and *CCEAE6A* were found over expressed in resistant populations [38].

To date, there is a lack of information available on the resistance status and mechanisms of *Ae*. *aegypti* in Laos and consequently, this is questioning the possible impact of resistance on vector control operations through the country. The objective of the present study was to assess the resistance status of *Ae*. *aegypti* exposed to different public health pesticides and to identify the underlying resistance mechanisms in resistant populations.

## Materials and methods

### Mosquito collections

**Fig 1:**
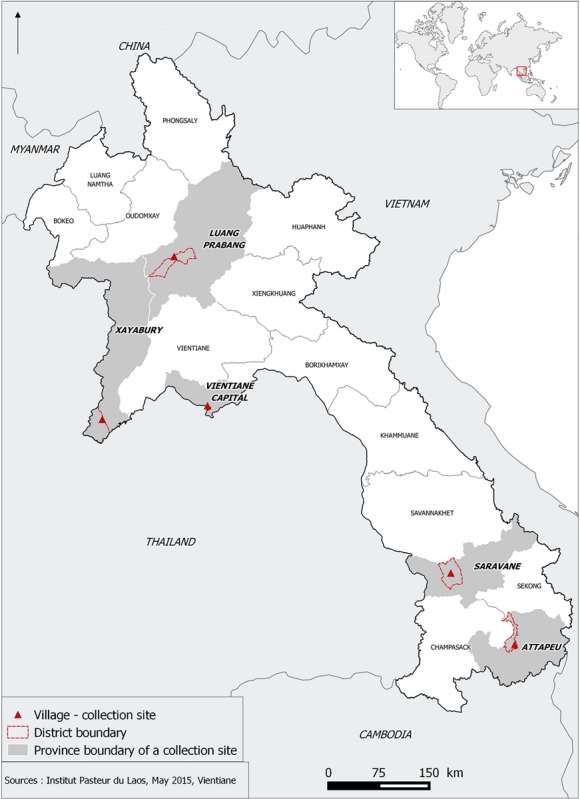
Locations of the mosquito collection sites. Source: MALVEC Project 2016, Institut Pasteur du Laos—IRD, SavGIS software.

During the rainy season of 2014, we sampled 11 populations from five different provinces In each province, we focused on the largest and most urbanized cities that have experienced increased dengue transmission or outbreaks in the last few years and where vector control was implemented during these episodes. *Aedes* larvae and pupae were collected in 11 villages belonging to five different provinces of Laos. Larvae were mainly collected in households and in temples. All the collection sites were geo-referenced (Table 1). Fig 1 shows the location of the collection sites in Laos. All samples were brought back to the laboratory and reared under controlled conditions (27 ± 2°C and 80 ± 10% relative humidity) until adults (F0 generation). After adult identification, mosquitoes were separated by species and *Ae*. *aegypti* specimens were kept for breeding following standardized techniques [42]. F1 larvae and adults were used for bioassays, with an insecticide susceptible reference (USDA) strain used as control. The USDA laboratory population is originating fromthe Center for Medical, Agricultural, and Veterinary Entomology, Gainesville, FL, U.S.A and was colonized continuously for 40 years at Kasetsart University, Bangkok, Thailand [43]. This strain was then colonized at the Institut Pasteur du Laos (IPL) before the experiments.

**Table 1:** List of the *Aedes aegypti* populations collected from the north to the south and their GPS coordinates. Population abbreviation names are used throughout the text.

Province	District	Village	Abbreviations	GPS coordinates	
Xayaboury	Borten	Taling	XBRTL	17.784729°N	101.170521°E
Luang Prabang	Luang Prabang	Khomkhuang Thatnoy Thongchaleun	LPBKKG LPBTNY LPBTCL	19.902775°N 19.531432°N 19.887366°N	102.156213°E 102.075364°E 102.132352°E
Vientiane Capital	Saythany Chanthabouly Sisattanak	Oudomphon Phailom Dongpalab kao-gnot*	VTEODP VTEPLM VTEDPL VTEIPL	18.125733°N 18.057037°N 17.988083°N 17.962684°N	102.665011°E 102.774993°E 102.605268°E 102.615035°E
Saravane	Lakhonepheng Vapi	Lakhonepheng Khonsaiy	SRVLKP SRVKS	15.485507°N 15.414079°N	105.403469°E 105.541816°E
Attapeu	Samakheexay	Xaysa-art	ATPXA	14.484109°N	106.501415°E

*VTEIPL strain, collected at the Institut Pasteur du Laos

### Bioassays

#### Larval bioassay

Larval bioassays were carried out according to WHO guidelines [44] using technical grades temephos (96.1% active ingredient [a.i.]), deltamethrin (99.7% a.i.), permethrin (98.1% a.i.), and DDT (99.4% a.i.) purchased from Sigma-Aldrich (Singapore). Even though pyrethroids are not used for larval treatment we tested them againt *Ae*. *aegypti* to obtain information of the larval resistance status that may reflect the adult resistance status. Bioassays were performed on late third- and early fourth-instar larvae of each population. Bioassay were performed in recipients containing 99 mL of distilled water and 1 mL of the insecticide tested at the desired concentration. Five replicates per concentration (25 larvae per replicate) and 5–8 concentrations in the activity range of each insecticide were used to determine the lethal concentrations (LC_50_ and LC_95_) and to compare them against the USDA strain. Control treatments consisted of 1% ethanol. Larval mortality was recorded after an exposure of 24 hours. For each bioassay, temperature was maintained at 27 ± 2°C and 80 ± 10% relative humidity with a 12-hour light: 12-hour dark photoperiod. Field populations were considered resistant to a given insecticide when their Resistance Ratios (RR) compared to the USDA strain showed confidence limits excluding the value 1. We considered resistance to be moderate when the RR values were between 2 and 5, and strong when the RR values were over 5 [44].

#### Adult bioassay

Adult bioassays were run using filter papers treated with diagnostic doses of deltamethrin (0.05%), permethrin (0.25%), DDT (4%), and malathion (0.8%) following WHO protocol [44]. Four batches of 25 non-blood-fed females (2–5 days old) were introduced into holding tubes and maintained for 60 minutes at 27 ± 2°C and a relative humidity of 80 ± 10%. Insects were then transferred into the exposure tubes and placed vertically for 60 minutes under subdued light. Mortality was recorded 24 hours after exposure and maintained in similar conditions of temperature and humidity. Following WHO criteria [44] a population was considered resistant if the mortality was below 90%, resistance was suspected when mortality ranged from 90% to 98% and a population was considered susceptible when mortality was over 98%.

#### Synergist study

Synergist bioassays using the specific enzyme inhibitors piperonyl butoxide (PBO), tributyl triphosphorotrithioate (DEF) and, diethyl maleate (DEM) were conducted to address the potential role of P450s, CCEs and GSTs in insecticide resistance. Larvae from one population of each province were exposed for 1 hour to the enzyme inhibitors PBO (1 mg/L), DEF (0.08 mg/L) and, DEM (1 mg/L) and then 24 hours to temephos following the same protocol described in larval bioassay section to measure the synergist ratios (SR). Adult mosquitoes were exposed 1 hour to sub lethal concentration of PBO (4%), DEF (8%) and DEM (8%) prior to the insecticides (malathion and permethrin) following the same WHO protocol for adult bioassays [44]. Mortality was recorded 24 hours after insecticide exposure. The synergist study on adults was performed after and independently (different mosquito batches) from the bioassays done without inhibitors.

#### Enzymatic activities of the detoxification enzymes

The levels of P450 monooxygenases (P450s), and the activities of carboxy/cholinesterases (CCEs) and glutathione S-transferases (GSTs) were assayed from single 3 days-old F1 females (n = 47) following microplate methods described by Hemingway [45] and Brogdon [46] on a spectrophotometer. Total protein quantification of mosquito homogenates was performed using Bradford reagent with bovine serum albumin as the standard protein [47] to normalize enzyme activity levels by protein content. For P450 assays, the optical density (OD) values were measured at 620 nm after 30 min incubation of individual mosquito homogenate with 200 mL of 2 mM 3, 3’, 5, 5’-tetramethylbenzidine dihydrochloride (TMBZ) and 25 mL of 3% hydrogen peroxide and the quantity was determined from cytochrome-c standard curve. Nonspecific α - and b-CCEs activities were assayed by 10 min incubation of mosquito homogenate in each well with 100 mL of 3 mM napthyl acetate (either α - or β -) at room temperature and the OD values were measured at 540 nm. The activity was determined from α - or β -naphtol standard curves. Glutathione-S-transferases activity was measured in the reaction containing 2 mM reduced glutathione and 1 mM 1-chloro-2,4-dinitrobenzene (CDNB). The reaction rates were measured at 340 nm after 20 min, and the activity was expressed in nmoles GSH conjugated/min/mg protein.

#### *Kdr* genotyping

We investigated the V1016G and 1534C *kdr* mutations because they were described in the literature as good markers of pyrethroid resistance in *Aedes* mosquitoes[31,48–50]. Unfortunately, we did not genotype the S989P mutation because at the time of the implementation of the study this mutation was not yet detected in the region.

Biological material consisted of DNA extracts from 11 populations for which individuals were extracted and genotyped for the 1016G and 1534C *kdr* mutations by qPCR. Each population contained 100 extracts: 50 for permethrin and 50 for DDT (25 dead and 25 live mosquitoes from the bioassays). Total number of extracts to proceed by qPCR was 1076.Total genomic DNA was extracted according to manufacturer’s instructions (Spin-column extraction method; NucleoSpin 96 virus, Macherey-Nagel, GmbH & Co. KG, Germany; protocol available at www.mn-net.com). Detection of the V1016G mutation and the F1534C mutation were implemented by qPCR based on the high resolution melting (HRM) curve developed by Saaverda-Rodriguez et al. [51] and Yanola et al. [52], respectively. Resistant and susceptible homozygotes for both mutations were used for controls. The mix used for the V1016G mutation was composed of ultrapure water, 3μL of EvaGreen Euromedex 5X for fluorescence, 4.5pmol of Gly1016 r/f, 0.7pmol of Val1016r primers (S1 Table) and 3μL containing 5 to 50 ng of DNA in a total volume of 15μL. The mix for the F1534C mutation contained 3μL of EvaGreen Euromedex 5X, 2pmol of each forward primer and 4 pmol of the reverse primer in a total volume of 15μL. Each reaction was performed in duplicate. Fluorescence data was analyzed by the Bio-Rad CFX Manager 3.0 software and the Precision Melt Analysis Software.

**Copy Number Variations of detoxification genes** The presence of CNVs affecting a set of candidate genes (S2 Table) encoding detoxification enzymes previously associated with insecticide resistance in SEA [32,37,53] was investigated in five populations representative of the whole country: LPBTCL, VTEODP, VTEDPL, VTEIPL and SRVLKP. Targeted genes consisted in the carboxylesterase, CCE-like (previously CCEae3A, AAEL023844) located in a cluster of multiple CCEs on chromosome 2, and four cytochrome P450s: CYP6BB2 and CYP6P12 located within a CYP6 cluster on chromosome 1 together with the CYP9J-like (AAEL014614) and CYP9J28 located within a large cluster of CYP9s on chromosome 3.

CNV were estimated for these genes in individual mosquitoes by quantifying their relative genomic DNA quantity by real-time quantitative PCR as compared to the USDA strain. For each population, 28 DNA samples previously used for *kdr* genotyping were analyzed (7 survivors and 7 dead from the DDT and permethrin bioassays). As no genetic association was expected between *kdr* mutations and metabolic resistance alleles, individuals were chosen in order to ensure a good representation of *kdr* genotypes present in each population. PCR amplifications were performed in duplicates on an iQ5 cycler (Bio-Rad) using specific primer pairs for each gene (S1 Table). Amplification reactions consisted of 3 μL gDNA template, 3.6 μL nuclease free water, 0.45 μL of each primer (10mM) and 7.5 μL of SYBR Green Supermix 2x (Bio-Rad). A dilution scale made from a pool of all gDNA samples was used for assessing PCR efficiency. The relative DNA quantity of each sample versus the USDA susceptible strain was calculated using the ΔΔCt method taking into account PCR efficiency [54] and the genes encoding CYP4D39 (AAEL007808) and a chloride channel (AAEL005950) for normalizing DNA quantity.

### Statistical analysis

For each strain and each insecticide, the dose mortality relationships were fitted by regression (P>0.05). Results were analyzed using the log-probit method of Finney (1971) using the Log dose Probit software (LdP) Line (Ehabsoft, Cairo, Egypt) to estimate the slope of regression lines and determine the 50% and 95% lethal concentration (LC_50_ and LC_95_, respectively) with 95% confidence intervals (CIs). For each bioassay, when control mortality was greater than 5% but less than 20%, then the observed mortalities were corrected using Abbott’s formula [55]:

The resistant ratios (RR_50_ and RR_95_) were obtained by calculating the ratio between the LC_50_ and LC_95_ of the wild and USDA strains. Synergist ratios (SR_50_ and SR_95_) were obtained by calculating the ratio between LC_50_ and LC_95_ with and without enzyme inhibitor.

Statistical comparisons of detoxification enzyme levels between USDA and the field populations were assessed with the Wilcoxon—Mann Whitney test in BiostaTGV (Institut Pierre Louis d'Epidémiologie et de Santé Publique UMR S 1136; https://biostatgv.sentiweb.fr/).

To assess the role of each mutation in permethrin and DDT resistance, we conducted a phenotype–genotype analysis by comparing the genotypic distribution of the V1016G and F1534C mutations between the dead and live mosquitoes. Comparisons were made using Fisher's exact test at 95% CI, in genepop software 4.2 (Laboratoire de Génétique et Environment, Université de Montpellier, http://genepop.curtin.edu.au/, [56,57]). Considering that the comparisons cannot be done with unexposed populations having fixed alleles (i.e. around 1 or 0), we selected populations having *kdr* allelic frequency for both mutations ranging from 0.3 to 0.7.

CNV analysis first consisted in testing for each gene the association between relative copy number values obtained from all individuals and their survival/death status to DDT or permethrin among and within populations using a Kruskall-Wallis test. Then individual relative copy number values were averaged for each population in order to test the association between CNV and insecticide resistance levels at both life stages (RR_50_ for larvae and survival rates for adults) using Pearson’s product moment correlation coefficient test.

## Results

### Bioassay

#### Larvae resistance levels

The insecticide resistance level to temephos, deltamethrin, permethrin, and DDT are presented in Table 2. All *Ae*. *aegypti* populations from Laos showed resistance to the larvicide temephos (see criteria above) with RR_50_ and RR_95_ between 1.55 and 6.5, respectively. Populations from Xayaboury (XBRTL), Vientiane Capital (VTEODP), Attapeu (ATPXA), and Saravane (SRVLKP) showed the highest resistance levels with (RR_50_ between 3 and 4). The population from Attapeu and Saravane showed RR_95_ > 5. All the populations showed strong resistance to deltamethrin and permethrin with RR_50_ between 6.25 and 17.5 except the population from Attapeu which showed a moderate resistance to permethrin (RR_50_ = 2). Mosquitoes from Vientiane were the most resistant to deltamethrin and permethrin (RR_50_ between 8 and 19). High resistance to DDT was measured with RR_50_ up to 171.7 in Xayaboury mosquitoes and the most resistant (RR_50_ between 94 and 124) mosquito populations were from Vientiane capital.

**Table 2:** Resistance status of *Aedes aegypti* larvae (reference and field strains) against temephos, deltamethrin, permethrin, and DDT.

Insecticide																												
	temephos							deltamethrin							permethrin							DDT						
Strain	Slope (± se)	LC_50_ (95% CI) (μg/L)	LC_95_ (95% CI) (μg/L)	RR_50_ *	RR_95_ *	χ^2^	p	Slope (± se)	LC_50_ (95% CI) (μg/L)	LC_95_ (95% CI) (μg/L)	RR_50_ *	RR_95_ *	χ^2^	p	Slope (± se)	LC_50_ (95% CI) (μg/L)	LC_95_ (95% CI) (μg/L)	RR_50_ *	RR_95_ *	χ^2^	p	Slope (± se)	LC_50_ (95% CI) (μg/L)	LC_95_ (95% CI) (μg/L)	RR_50_ *	RR_95_ *	χ^2^	p
USDA	4.6 (0.3)	2.9 (2.7–3.1)	6.6 (5.8–7.6)	-	-	3.7	0.59	1.8 (0.2)	0.08 (0.07–0.1)	0.66 (0.43–1.2)	-	-	0.6	0.7	10 (0.8)	1.1 (1.1–1.2)	1.6 (1.6–1.7)	-	-	3	0.56	2.6 (0.2)	4.6 (4.1–5.1)	19.6 (16–25.8)	-	-	7.7	0.10
XBLTL	2.2 (0.14)	13.5 (12.2–15.2)	73.8 (57.5–101)	**3.62**	**3.6**	1.6	0.44	3.3 (0.3)	0.6 (0.55–0.66)	1.9 (1.6–2.4)	**7.5**	**2.9**	3.9	0.3	4 (0.3)	10.3 (9.6–11)	26.3 (23.2–31.1)	**9.4**	**16.4**	5.8	0.21	4.8 (0.6)	790 (728–891)	1750 (1402–2519)	**171.7**	**89.3**	4.1	0.39
LPBKKG	4.2 (0.2)	4.5 (4–5)	11.1 (10–13.4)	**1.55**	**1.68**	9.9	0.02	3.4 (0.2)	0.57 (0.54–0.6)	1.75 (1.57–2.01)	**7.1**	**2.6**	5	0	-	-	-	-	-	-	-	3.3 (0.5)	112 (76–139)	359 (323–418)	**24.4**	**18.3**	0.2	0.88
LPBTNY	5.5 (0.3)	5.6 (5.3–5.9)	11.7 (10.8–12.9)	**1.93**	**1.77**	5.5	0.14	3.4 (0.4)	0.92 (0.86–1.02)	2.82 (2.27–3.81)	**11.5**	**4.3**	3.1	0.4	-	-	-	-	-	-	-	4.7 (0.4)	382 (359–404)	856 (769–985)	**83**	**43.7**	4.8	0.3
LPBTCL	5.1 (0.3)	6.7 (6.5–7.1)	14.1 (12.7–16.2)	**2.31**	**2.14**	0.3	0.96	2.7 (0.3)	0.96 (0.87–1.09)	3.93 (2.91–6.16)	**12**	**5.9**	0.6	1	4.2 (0.32)	9.2 (8.7–9.8)	22.7 (20–26.4)	**8.4**	**14.2**	2	0.85	1.4 (0.2)	87 (52–118)	1380 (958–2547)	**18.9**	**70.4**	3.5	0.63
VTEODP	4.2 (0.5)	7 (6.4–7.8)	17.2 (13.8–24.4)	**3.5**	**2.61**	4.5	0.10	4.8 (0.4)	1.1 (1–1.2)	2.26 (1.9–2.8)	**13.8**	**3.4**	1.9	0.4	3.6 (0.3)	13.5 (10.4–19.5)	38.8 (37.9–123)	**13.5**	**24.3**	12	0.01	5.2 (0.4)	435 (410–457)	901 (833–995)	**94.6**	**45.9**	5.7	0.34
VTEPLM	4.8 (0.5)	5.4 (4.9–5.9)	11.9 (10.6–14)	**1.86**	**1.8**	0.3	0.88	3.8 (0.6)	1.5 (1.2–1.9)	3.9 (2.7–8)	**18.8**	**5.9**	1.1	0.8	-	-	-	-	-	-	-	3.9 (0.5)	571 (518–641)	1479 (1185–2076	**124.1**	**75.4**	3.1	0.21
VTEDPL	4.5 (0.3)	6.4 (5.9–6.9)	18.8 (15.6–24.7)	**2.21**	**2.85**	4.5	0.21	2.8 (0.3)	0.65 (0.59–0.72)	2.45 (1.9–3.5)	**8.1**	**3.7**	5.8	0.3	-	-	-	-	-	-	-	3.1 (0.3)	461 (421–503)	1587 (1310–2074)	**100.2**	**80.9**	0.9	0.82
VTEIPL	6.6 (0.5)	6.6 (6.2–6.9)	11.6 (10.5–13.5)	**2.28**	**1.76**	5.3	0.07	2.6 (0.2)	1 (0.91–1.11)	5.2 (3.9–7.5)	**12.5**	**7.9**	2.6	0.6	4.8 (0.45)	16.7 (15.2–19.5)	36.5 (32–57.7)	**15.2**	**22.8**	13	0.03	3.8 (0.3)	441 (404–476)	1180 (1034–1403)	**95.9**	**60.2**	2.0	0.58
SRVLKP	2.5 (0.3)	9.7 (8.3–11.1)	43.1 (33.4–63.7)	**3.34**	**6.5**	1.6	0.66	4.3 (0.3)	1.4 (1.3–1.6)	6.8 (5.1–10)	**17.5**	**10.3**	11	0.1	5.1 (0.4)	10.6 (10–11.2)	22.2 (19.9–25.7)	**9.6**	**13.9**	3.8	0.58	3.6 (0.4)	332 (301–361)	941 (801–1186)	**72.2**	**48**	6.7	0.15
SRVKS	3.8 (0.3)	8.8 (8.2–9.7)	24.2 (19.9–31.9)	**2.38**	**4.24**	1.3	0.53	3 (0.2)	0.5 (0.4–0.6)	1.6 (1.4–1.9)	**6.25**	**2.4**	8	0.2	-	-	-	-	-	-	-	3.4 (0.3)	44 (29–58)	135 (125–300)	**9.7**	**6.9**	24.9	0.0001
ATPXA	2.9 (0.8)	9.9 (6.9–33)	36.7 (36.2–61)	**3.41**	**5.56**	0.5	0.78	3.7 (0.3)	0.5 (0.4–0.6)	1.4 (1.2–1.9)	**6.25**	**2.3**	9.8	0.1	3.9 (0.5)	2.2 (1.5–2.5)	5.7 (5.1–9.6)	**2**	**3.6**	9.9	0.04	2.4 (0.2)	35 (25–47)	174 (144–334)	**7.6**	**8.9**	22.9	0.0004

LC = Lethal concentration, RR = Resistant Ratio, CI = confidence interval. Significant RR are shown in bold.

### Adult resistance levels

**Fig 2:**
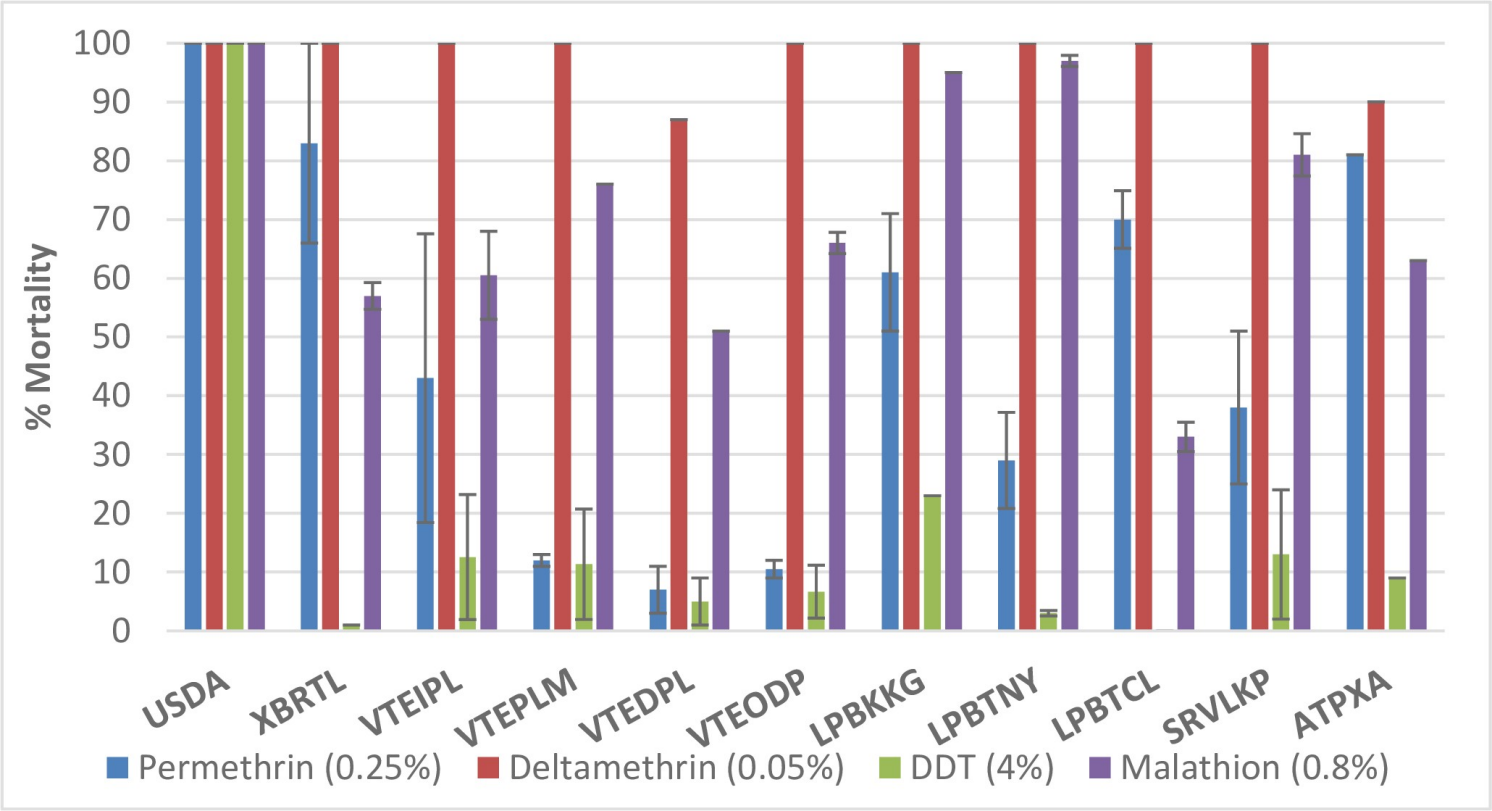
Mortality rates and 95% confidence intervals in WHO tube tests of wild adult *Aedes aegypti* populations from Laos. USDA: susceptible reference strain.

As expected, the USDA strain showed full susceptibility to the four insecticides tested (100% mortality, Fig 2). Almost all populations tested were highly resistant to DDT, permethrin and malathion with mortality rates varying from 0 to 31%, from 7 to 83%, and from 33 to 98%, respectively. All populations were susceptible to deltamethrin (> 98% mortality) except for the VTEDPL and ATPXA populations, which showed moderate resistance (~90% of mortality). All the populations tested against malathion were resistant (mortality < 80%) or showed suspected resistance (mortality between 90 and 98%) to this insecticide.

### Effect of detoxification enzymes on resistance

#### Larvae

In the susceptible strain, temephos toxicity was significantly increased (for SR_50_ but not for SR_95_) in the presence of the detoxification enzyme inhibitor DEF. There was a significant but low decreased temephos toxicity in the presence of PBO and DEM (SRs < 1). Except for VTEIPL and LPBTCL populations, the toxicity of temephos increased significantly in all populations in the presence of detoxification enzyme inhibitors (Table 3). The levels of resistance to temephos was reduced for all the population particularly in the presence of DEF compared to DEM and PBO indicating the major role of CCEs in resistance to organophosphates.

**Table 3:** Insecticidal activity of temephos against *Aedes aegypti* larvae (reference strain and field populations) with and without inhibitors.

Strain	Larvicides	Slope (± se)	LC_50_ (95% CI) (μg/L)	LC_95_ (95% CI) (μg/L)	χ^2^	p	SR_50_ *	SR_95_ *
USDA	temephos	4.6 (0.3)	2.9 (2.7–3.1)	6.6 (5.8–7.6)	3.7	0.59	-	-
	temephos + DEF	3.2 (0.2)	1.3 (0.9–1.7)	4.2 (3.4–8.1)	11.9	0.0075	**2.23**	1.57
	temephos + DEM	8.1 (0.8)	5.3 (5.1–5.6)	8.5 (7.6–9.8)	5.7	0.057	**0.54**	**0.78**
	temephos + PBO	5 (0.4)	4.3 (3.6–5.2)	9.1 (8.2–16.3)	8.7	0.034	**0.67**	**0.73**
SRVLKP	temephos	2.5 (0.3)	9.7 (8.3–11.1)	43.1 (33.4–63.7)	1.6	0.66	**-**	**-**
	temephos + DEF	3.6 (0.4)	3.2 (2.9–3.6)	9.4 (7.5–13)	0.24	0.62	**3.03**	**4.59**
	temephos + DEM	5.9 (0.8)	7.5 (7–8)	14.1 (12.2–17.9)	0.006	0.94	**1.29**	**3.06**
	temephos + PBO	6.2 (0.5)	7.3 (6.8–7.8)	13.4 (12–15)	0.26	0.61	**1.33**	**3.21**
XBRTL	temephos	2.2 (0.14)	13.5 (12.2–15.2)	73.8 (57.5–101)	1.6	0.44	**-**	**-**
	temephos + DEF	1.8 (0.1)	3.3 (2.7–3.9)	24.9 (19.8–33.8)	8.8	0.12	**4.1**	**2.96**
	temephos + DEM	5.5 (0.5)	11 (10.3–11.7)	21.9 (19.5–25.7)	6.4	0.09	**1.23**	**3.37**
	temephos + PBO	4.3 (0.4)	10.1 (9.4–10.9)	24.5 (21.3–29.8)	6.4	0.09	**1.34**	**3.01**
LPBTCL	temephos	5.5 (0.4)	6.7 (6.5–7.1)	14.1 (12.7–16.2)	0.31	0.95	-	-
	temephos + DEF	3.8 (0.4)	2.1 (1.9–2.3)	5.8 (4.9–7.2)	5.5	0.14	**3.19**	**2.43**
	temephos + DEM	5.5 (0.8)	5.2 (4.9–5.6)	8.7 (7.5–11.5)	0.026	0.87	**1.29**	**1.66**
	temephos + PBO	7.4 (1.3)	5.7 (5.3–6.4)	11.4 (9.2–16.7)	0.15	0.92	**1.17**	1.24
VTEIPL	temephos	6.6 (0.5)	6.6 (6.2–6.9)	11.6 (10.5–13.5)	5.3	0.07	**-**	**-**
	temephos + DEF	3.7 (0.3)	3 (2.7–3.3)	8.3 (7.1–10.2)	3.4	0.49	**2.2**	**1.4**
e	temephos + DEM	6.5 (0.9)	5,4 (5.1–5.8)	9.7 (8.3–12.5)	1.5	0.48	**1.22**	1.2
e	temephos + PBO	7.6 (0.9)	5.6 (5.3–6)	9.2 (8.2–11)	0.7	0.4	**1.18**	**1.26**
ATPXR	temephos	2.9 (0.8)	9.9 (6.9–33)	36.7 (36.2–61)	0.5	0.78	**-**	**-**
	temephos + DEF	3.7 (0.3)	3 (2.7–3.3)	8.3 (7.1–10.2)	3.3	0.5	**3.3**	**4.42**
	temephos + DEM	6.4 (0.8)	5.4 (5.1–5.8)	9.7 (8.3–12.5)	1.5	0.48	**1.83**	**3.78**
	temephos + PBO	7.6 (0.9)	5.6 (5.3–6)	9.2 (8.2–11)	0.7	0.4	**1.77**	**3.99**

*LC = Lethal concentration. CI = Confidence Interval. SR = Synergist ratio, Significant SR are shown in bold.

#### Adults

**Fig 3:**
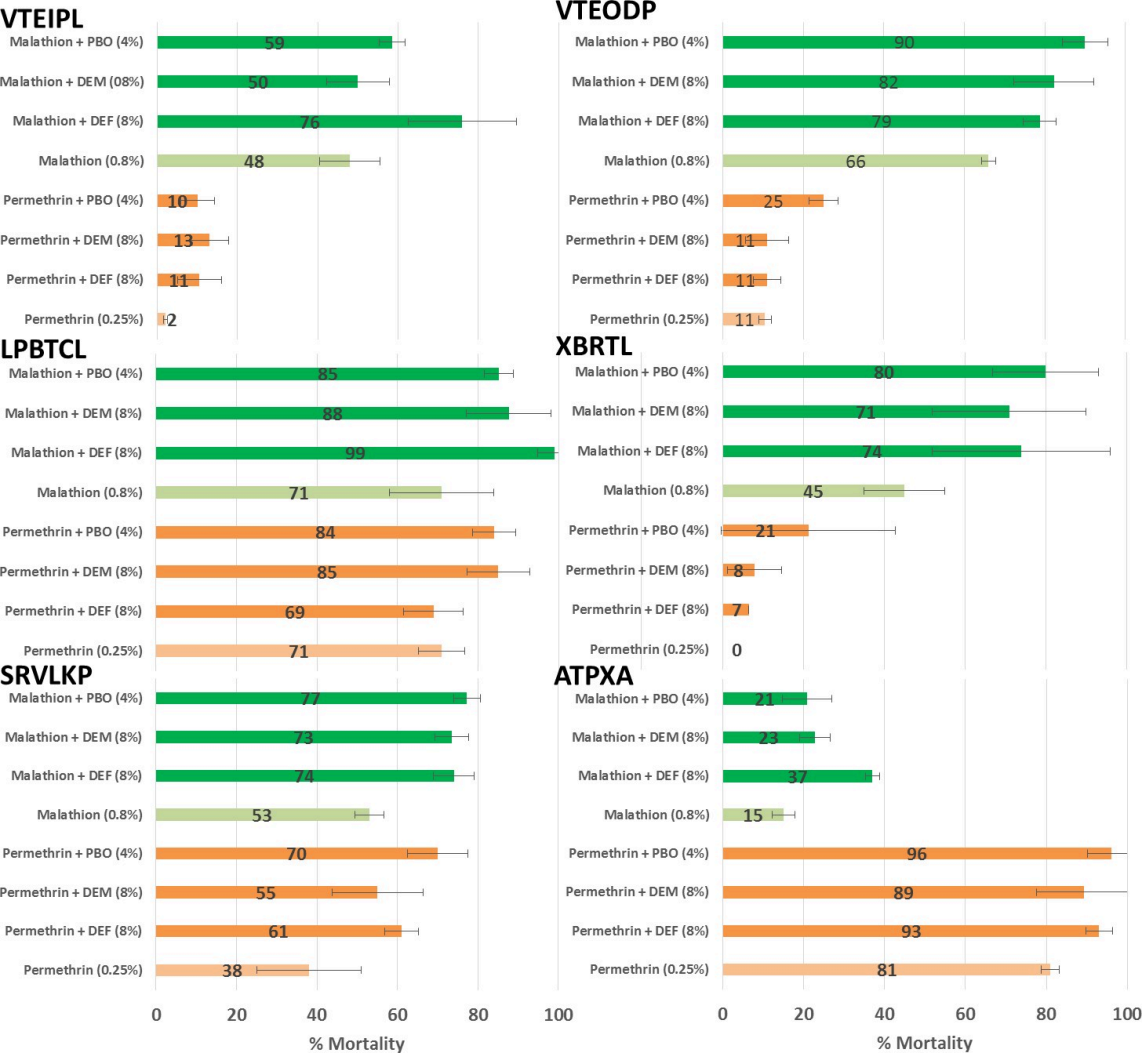
Mortality (%) and standard deviation of adult *Aedes aegypti* after exposure to permethrin (0.25%) and malathion (0.8%) with and without inhibitors (DEF: 8%, DEM: 8%, and PBO: 4%).

We did not observe full insecticide susceptibility recovery after pre-exposure to synergists except in one population (LPBTCL) that was exposed with malathion and DEF synergist (from 71% to > 98% mortality, Fig 3). For several populations tested, the toxicity of permethrin and malathion increased in the presence of inhibitors, indicating the involvement of P450s, CCEs and GSTs in the resistance to these two insecticides. Specifically, five out of the six populations (VTIPL, VTODP, LPBTCL, SRVLKP, and ATPXA) exhibited higher susceptibility to malathion in the presence of DEF indicating the involvement of CCEs in the resistance. Several populations showed a higher susceptibility to malathion in the presence of PBO (VTEODP, XBLTL, and SRVLKP) and DEM (VTEODP, SRVLKP, and ATPXA) indicating a potential role of P450s and GSTs in the phenotypic resistance, respectively. All populations (except XBRTL) showed a significantly increased susceptibility to permethrin after PBO pre-exposure. We observed a partial recovery of susceptibility with both DEM and DEF synergists (VTEIPL and XRBTL) suggesting a possible additional role of GSTs and CCEs in permethrin resistance.

### Biochemical assays

**Fig 4:**
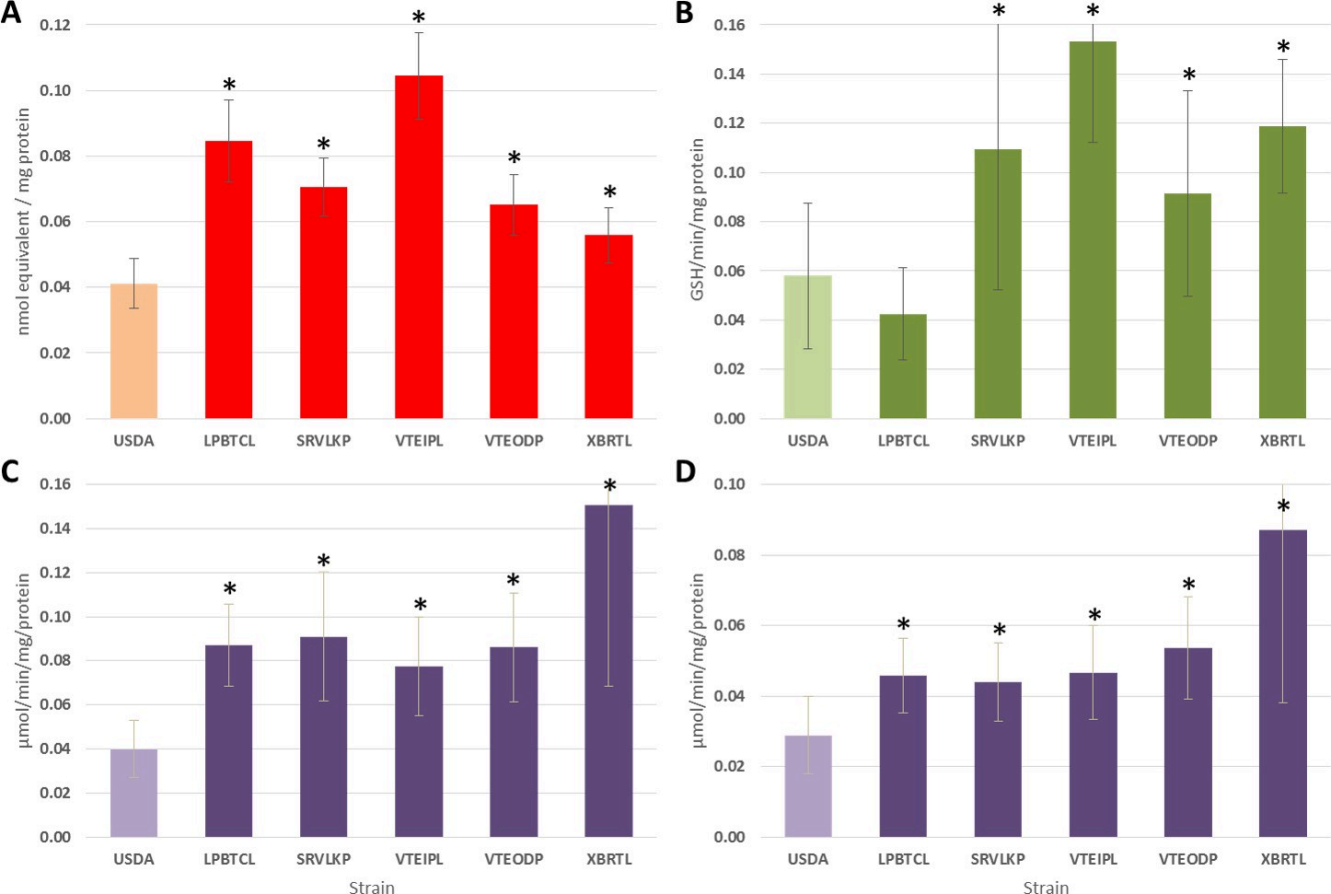
Activity of detoxification enzymes and 95% Confidence Interval in field caught *Aedes aegypti* populations in comparison to the susceptible reference strain (USDA): cytochrome P450 monooxygenases (P450s; A), Glutathione-S transferases (GSTs; B), Esterases (α and β-CCEs; C and D). Sample sizes are 47 specimens/population. An asterisk (*) denotes significantly higher levels of detoxifying enzyme compared to the susceptible reference strain USDA.

Compared to the USDA strain, all populations (except LPBTCL for GST) showed a significantly higher constitutive detoxification enzymes level/activities for the P450s, GSTs, α-CCE and β-CCE enzymes tested (*P <* 0.01, Wilcoxon—Mann Whitney, Fig 4).

### Alleles and haplotypes frequencies of kdr mutations

**Fig 5:**
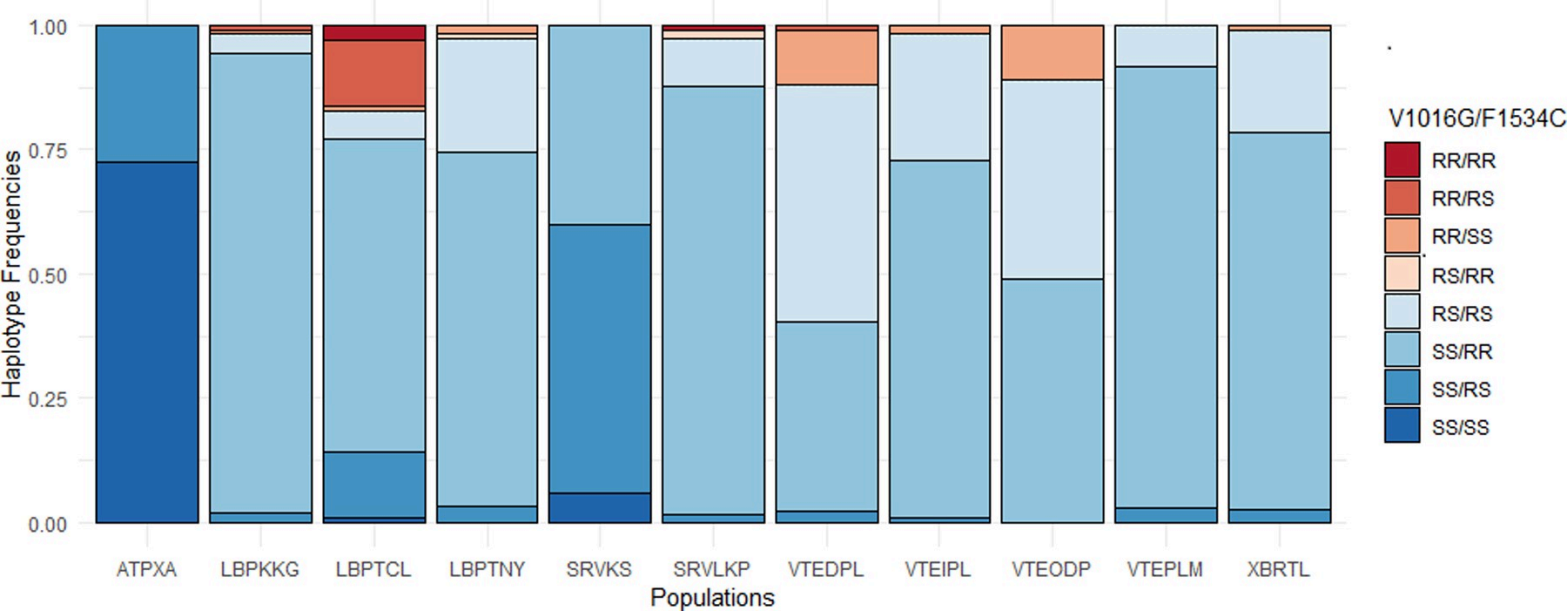
Haplotypes frequencies of V1016G and F1534C *kdr* mutations in *Aedes aegypti* populations from Laos.

A total of 1,076 *Ae*. *aegypti* females from 11 field-caught populations were tested by real-time PCR to detect the presence of the V1016G and F1534C *kdr* mutations (Table 4). The V1016G mutation was found at low and variable frequencies from 0 (SRVKS and ATPXA) to 0.36 (VTEDPL). The 1534C mutation was found at high frequency in all populations (> 0.6) except at ATPXA where the prevalence was low (F(R) = 0.14). Significant differences in the frequency of the 1016G and 1534C alleles were found between populations (*P* < 0.05). All populations were at Hardy-Weinberg Equilibrium except LPBKKG (*P* = 0.0043) and LPBTCL (*P* < 0.001) for the V1016G mutation (S3 Table). We also checked for linkage disequilibrium between V1016G and F1534C in all populations (S3 Table). All but two populations, SRVKS and ATPXA, had a non-random negative association of alleles at the two *kdr* loci (*P* < 0.001). In other words, individuals exhibiting RR genotypes for the 1016G were mostly SS for the 1534C and *vice versa* (Fig 5). Only 4 mosquitoes out of 1,076 were double homozygote resistant for both 1534C and 1016G. We did not find any association between the frequency of *kdr* mutations (for both F1534C and 1016G and haplotypes) and the survival rate to DDT and permethrin (*P* > 0.05; S4 Table).

**Table 4:** Genotype distribution and allelic frequency of the V1016G and F1534C *kdr* mutations.

	V1016G				F1534C			
POPULATION	SS	RS	RR	F(R)	SS	RS	RR	F(R)
**VTEIPL**	86	30	2	0.14	2	31	85	0.85
**VTEDPL**	34	40	10	0.36	12	43	32	0.62
**VTEODP**	53	43	12	0.31	12	43	53	0.69
**VTEPLM**	90	8	0	0.04	0	11	87	0.94
**XBRTL**	91	24	1	0.11	1	27	88	0.88
**LPBKKG**	102	4	2	0.04	1	7	100	0.96
**LPBTCL**	81	6	18	0.20	2	34	69	0.82
**LPBTNY**	87	28	2	0.14	2	31	84	0.85
**SRVLKP**	101	13	1	0.07	0	13	102	0.94
**SRVKS**	50	0	0	0.00	3	27	20	0.67
**ATPXA**	50	0	0	0.00	37	14	0	0.14

### Copy Number Variations of detoxification genes

**Fig 6:**
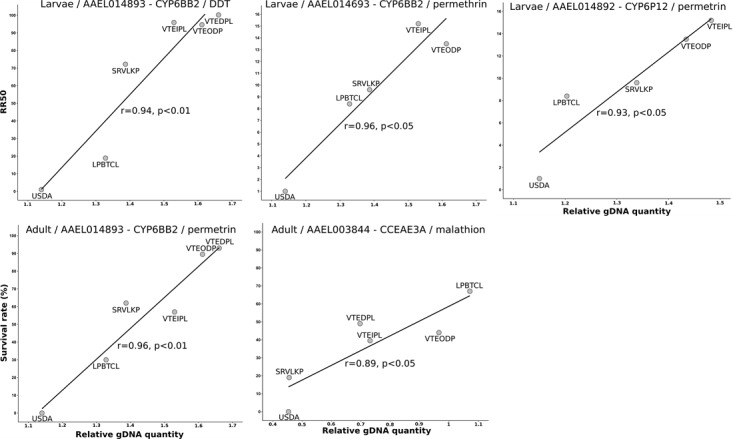
Significant positive correlations identified CNV of detoxification genes and population resistance levels. Resistance levels are expressed as RR_50_ for larvae and % survival to WHO insecticide test for adults.

CNV were first analyzed at the individual level by comparing relative gene copy number between the dead and survivors exposed to DDT or permethrin in each population. Although some populations showed an elevated average higher gene copy number as compared to the USDA population, no significant association was found for any gene between gene copy number and survival to insecticide within and among populations. However, when copy number values were averaged for each population (including both the dead and survivors) and tested against population resistance levels (RR_50_ for larvae and survival rate to WHO test for adults), significant positive associations were found for three detoxification genes (Fig 6). CYP6BB2 gene copy number was correlated to both DDT and permethrin resistance in larvae and to permethrin resistance in adults. CYP6P12 gene copy number was correlated with permethrin resistance in larvae. Finally, CCEAE3A gene copy number was significantly positively correlated to malathion resistance in adults.

Because individuals used for CNV analysis were also genotyped for the presence of *kdr* mutations, the association between any particular *kdr* haplotype mutation and CNV affecting detoxification genes was tested. As expected, because CNV loci are distant from *kdr* loci, no significant association was found between *kdr* haplotypes and CNV for any gene.

## Discussion

The purpose of this study was to evaluate the insecticide resistance status of *Ae*. *aegypti* populations in several provinces throughout northern to southern areas of Laos. Insecticides representing the major families of insecticide used for vector control and (OC, OP, PYR) were tested against larvae and adult mosquitoes following WHO protocols. The possible insecticide resistance mechanisms involved were investigated using both biochemical and molecular assays and the association between gene copy number variations and resistance was investigated for a set of detoxification enzymes previously associated with resistance.

### Insecticide resistance levels in Laos and implication for public health

The use of temephos as larvicide has been broad and continuous for 30 years in Laos and have favored the selection of resistant *Aedes* sp. populations across the country. The resistance levels to this larvicide were evaluated in *Ae*. *aegypti* populations for the first time and our study showed that the populations tested were moderately resistant. Additionally, recent studies showed that *Ae*. *albopictus* populations, the secondary dengue vector in the country, were also resistant to temephos [58]. These results suggest that the use of alternative insecticides with different mode of action is urgently needed for the larval control of *Aedes* sp. in Laos. A semi-field trial implemented at the Institut Pasteur du Laos (IPL; VTEIPL strain) showed that *Bacillus thuringiensis israelensis* (*Bti*, biolarvicide) and diflubenzuron (insect growth regulator) formulations remain effective for extended periods (i.e. 28 weeks) in the containers widely used in Vientiane households for water storage [59]. Based on our findings, Lao Public Health authorities have recently modified their National Strategic Plan (NSP) to fight against dengue and have recommended the use of these alternative insecticides in yearly rotation (effective in 2019). The aim of this insecticide resistance management strategy is to preserve the efficacy (and susceptibility) of the few new alternative larvicides available for the control of dengue in the country.

Our study showed that resistance of adults to the OP malathion was widespread in Laos although resistance levels were heterogeneous across geographic regions. Our larvicide (temephos) findings showed that the use of OPs for vector control programs in Laos is no longer recommended. Furthermore, high levels of malathion resistance were detected in *Ae*. *albopictus* populations in Vientiane Capital and Luang-Prabang city in both rural and urban areas [58]. Malathion fogging treatments were used in the 1990’s and were progressively replaced by the use of PYRs in the 2000’s. Worldwide, resistance to malathion has been reported, and in several countries, this resulted in the replacement by other insecticides such as PYRs [60–62]. Unfortunately, PYR resistance in *Aedes* sp. mosquitoes is established in many countries [17] and our results in Laos reconfirm this trend. All the adult populations tested were highly resistant to permethrin and two of them, Attapeu and Vientiane Capital populations, were also resistant to deltamethrin. Similarly, *Ae*. *albopictus* mosquitoes showed high resistance levels to PYR in Laos [58]. All the populations tested against DDT were highly resistant even though this insecticide is no longer used for vector control. It is possible that *Aedes* mosquito populations are still exposed to DDT in Laos through illegal use and persistence in the environment [63], hence exerting constant selection pressure by maintaining DDT resistant alleles, such as *kdr* mutations, and P450s/GSTs resistant genes. These resistance mechanisms may confer selective advantages, such as cross-resistance to commonly-used PYR [24–26]. As already mentioned above, our study showed that most of the *Ae*. *aegypti* populations are still susceptible to the adulticide deltamethrin. However, we used the previous discriminating concentration of 0.05% recommended by WHO for *Aedes* mosquitoes, the tentative discriminating dose recommended for *Aedes* is now 0.03% [44] so we may have underestimated the phenotypic resistance to deltamethrin in our study. This hypothesis was confirmed with the larval bioassays results that show that all the populations tested were resistant to deltamethrin (Table 2).

### Knock down resistance mutations

Real-time PCR revealed that the 1016G and 1534C *kdr* alleles were present in *Ae*. *aegypti* in Laos at various frequencies. The population from Attapeu (ATPXA) had the lowest *kdr* frequencies for both mutations (F(R) = 0% for 1016G and = 14% for 1534C) and was also the most susceptible against PYR among all populations tested. The synergist study showed that resistance was partially recovered in the presence of synergists (<15%), suggesting that both enzymatic mechanisms and *kdr* mutations may play a role in the resistance to PYR in this population. However, for all the populations tested, no correlation was found between the presence of mutant alleles for both mutations and the survival rates of mosquitoes to DDT and permethrin, but several confounding factors may explain the outcomes. First, the metabolic-resistance background of live and dead mosquitoes was not taken into account in the genotype/phenotype association studies. It is likely that P450 genes associated with permethrin and deltamethrin resistance may have interfered with the survival rates of mosquitoes regardless of the *kdr* haplotype. Furthermore, a negative genetic association between the 1016 and 1534 *kdr* locus was found, indicating that the two genes are not independent from each other. The non-random association of the V1016G and F1534C haplotypes may have then interfered with the distribution of genotypes among dead and alive. The fact that only four mosquitoes (of 1,076) were found homozygote resistant for both 1534C and 1016G confirm this trend and suggest a high genetic cost associated with the double mutants. Similar results were reported in *Aedes* populations from Thailand and Myanmar [21,30]. Nevertheless, both mutations have been associated with PYR resistance in *Ae*. *aegypti* in SEA but with a contrasted pattern in their geographic frequencies distribution and correlation with insecticide resistance. For example, in Thailand, Malaysia, and Vietnam the 1534C allele was found at high frequency and 1016G at low frequency in populations resistant to PYRs, thus corroborating our results [31,52,64]. Other studies showed the opposite pattern in Thailand, southern China, and Myanmar [21,22,65]. As suggested by Kawada et al. [21], it possible that the use of DDT and permethrin (type I pyrethroid) and the use of deltamethrin (type II pyrethroid) favor the selection of F1534C and V1016G mutations, respectively. Wuliandari et al. [66] showed that the V1016G mutation was also associated with resistance to type II pyrethroids. The low frequency of this mutation in Laos might explain the low levels of resistance to deltamethrin in contrast to the high levels of permethrin resistance and high frequency of the F1534C substitution found in our study.

Several studies showed that another mutation in the sodium channel (S989P), if associated with the V1016G mutation can increase the resistance levels to PYR in *Ae*. *aegypti* [21,22,67], especially in a triple mutant 989P/1016G/1534C haplotype, which can engender extreme resistance [29]. However, triple mutants have been detected at low frequency in different Asian locations and monitoring of the occurrence and frequency of these association is a high priority [17]. The 989P/1016G/1534C heterozygote genotype confers moderate resistance to deltamethrin and may present an important step in the evolution of high-level resistance or a genotype providing benefit across different PYRs classes [33]. Recently, it has been demonstrated that a new mutation (V410L) alone or associated with F1534C confers resistance to both type I and II pyrethroid in Brazil [28]. The presence of this mutation should be investigated in Asia.

### Role of metabolic resistance mechanisms

Brooke and colleagues [68] early showed that resistance could be multigenic, and that the *kdr* genotype might not fully explain all the variance in phenotype. As discussed above, we assume that the absence of correlation between *kdr* mutations and survival rates to DDT and permethrin may also be explained by the co-occurrence of metabolic mechanisms and target site-mutations at the population level without any genetic link between them.

In our study, increasing mortality rates in larvae with temephos after pre-exposure to DEF suggest that CCEs are playing a predominant role in the observed resistance. Some reports based on routine biochemical assays in different *Ae*. *aegypti* field populations also supported the role of CCEs in OP resistance [39,69,70]. Furthermore, previous reports based on molecular tools correlated temephos resistance with CCEs over transcription [38]. In the adults, we observed a partial recovery of susceptibility after pre-exposure of the three synergists for both malathion and permethrin. For malathion, the synergist action of DEF indicates elevated CCEs activities thus showing that CCE genes are playing a major role in the resistance observed. However, mortality rates also increased with the use of DEM and PBO suggesting that other detoxification enzymes such as GSTs and P450s may also contribute to OP resistance. For deltamethrin, the effect of PBO confirms that the overexpression of P450 genes is playing an important role in the resistance although we cannot exclude that other detoxification enzyme families such as CCEs and GSTs may contribute to resistance. Our biochemical assays confirmed these results since most populations tested presented higher GSTs and CCEs activities and higher amounts of P450s compared to the susceptible strain (Fig 4). Overall these results confirm the important role of metabolic resistance in the resistance to OP, PYR, and DDT in *Ae*. *aegypti* in Laos as previously reported in the region [17]. Although classic biochemical and synergistic assays provide a first assessment of the presence of metabolic resistance, quantifying its importance versus other resistance mechanisms requires additional work.

### Gene copy number variations associated with metabolic resistance

At the individual level, CNV of candidate genes were not found significantly associated to insecticide survival for both insecticides. This was expected for DDT as no DDT marker was included in the candidate gene set but it was not expected for permethrin for which targeted detoxification genes have frequently been involved in pyrethroid resistance. Indeed, CYP6BB2 and CYP9J28 are known to encode cytochrome P450s able to degrade permethrin in *Ae*. *aegypti* and CNV affecting these genes have previously been associated with pyrethroid resistance [32,37,53,71]. In the present case, this absence of association may be explained by the low number of individual mosquitoes tested for each population/insecticide but also by the importance of *kdr* mutations in the ability of mosquitoes to survive high doses of permethrin and DDT. However, analyses performed at the population level allowed us to detect a significant correlation between permethrin resistance (adult and larvae) and CYP6BB2 gene copy number. This result suggests that although CYP6BB2 duplication was only detected in South America and that CYP6BB2 over-expression was not caused by a gene duplication in a pyrethroid-selected strain from Singapore [32,37,41], this duplication may still occur in South-East Asia. Whether CYP6BB2 up-regulation, genomic duplication, or both mechanisms can be selected in pyrethroid-resistant populations and be used as pyrethroid resistance markers in Souht-East Asia deserve further work. Finally, a strong correlation was detected in adults between malathion resistance, and CCEAE3A gene copy number. This suggests that this esterase previously shown to sequester and metabolize temephos [72,73], may also confer malathion resistance in Laos. Given the very low probability of occurrence of the Ace1 mutation conferring OP resistance in *Ae*. *aegypti* [74], the detection of CCEAE3A gene duplication represent a promising tool to track OP resistance in South-East Asia. Overall, although CNV affecting detoxification genes show a good potential to be used as DNA-based metabolic resistance markers in the field, their association with resistance to different insecticides and their importance in the overall resistance phenotype need to be confirmed.

### Conclusion

Overall, insecticide resistance in *Ae*. *aegypti* populations from Laos are concerning because of the lack of insecticides available for vector control, particularly for adult mosquitoes. Recently, the Lao government adopted a new strategy to deploy alternative larvicides with different modes of action in a yearly rotation. In the long term, this will help to maintain an effective vector control to prevent dengue and chikungunya transmission while preserving the susceptibility of available public health pesticides.

Our study suggests that both *kdr* mutations and metabolic resistance mechanisms are present in Laos but their impact on the resistance may differ in proportion at the population or individual level. Molecular analyses suggest that CNV affecting CCEAE3A previously associated with temephos resistance is strongly associated with malathion resistance while CNV affecting the two P450s, CYP6BB2 and CYP6P12, are associated with pyrethroid and possibly DDT resistance. The development of new tools to detect and better understand IR mechanism in *Ae*. *aegypti* is crucial to improve vector control and insecticide resistance monitoring.

## Supporting information

S1 Table List of the primers used for kdr and CNV detection. (PDF)

S2 Table Genes targeted for CNV analysis. (PDF)

S3 Table Kdr analysis, crude data from Genepop. (PDF)

S4 Table V1016G and F1534C genotypic differentiation and haplotype differentiation between dead and live mosquitoes for DDT and permethrin. (PDF)

## References

[1] WHO. Dengue Guidelines for Diagnosis, Treatment, Prevention and Control Special Programme for Research and Training in Tropical Diseases, editor. Geneva: World Health Organization; 2009.

[2] Lao M, Caro V, Thiberge J-M, Bounmany P, Vongpayloth K, Buchy P, et al Co-Circulation of Dengue Virus Type 3 Genotypes in Vientiane Capital, Lao PDR. Coffey LL, editor. PLoS ONE. 2014;9: e115569 10.1371/journal.pone.0115569 25551768 PMC4281081

[3] Vallée J, Dubot-Pérès A, Ounaphom P, Sayavong C, Bryant JE, Gonzalez J-P. Spatial distribution and risk factors of dengue and Japanese encephalitis virus infection in urban settings: the case of Vientiane, Lao PDR. Tropical medicine & international health. 2009;14: 1134–1142. 19563430 10.1111/j.1365-3156.2009.02319.x

[4] Fukunaga T, Phommasak B, Bounlu K, Saito M, Tadano M, Makino Y, et al Epidemiological situation of dengue infection in Lao PDR. Tropical Medicine. 1994;35: 219–227.

[5] Bounlu K, Tadano M, Makino Y, Arakaki S, Kanamura K, Fukunaga T. A seroepidemiological study of dengue and Japanese encephalitis virus infections in Vientiane, Lao PDR. Japanese Journal of Tropical Medicine and Hygiene. 1992;20: 149–156.

[6] Makino Y, Saito M, Phommasack B, Vongxay P, Kanemura K, Pothawan T, et al Arbovirus Infections in Pilot Areas in Laos. Trop Med. 1994;36: 131–139.

[7] WHO. Dengue situation update [Internet]. 2019. Available: http://www.wpro.who.int/emerging_diseases/DengueSituationUpdates/en/

[8] Halstead SB. Mosquito-borne Haemorrhagic Fevers of South and South-East Asia. Bulletin of the World Health Organization. 1966;35: 3–15. 5297536 PMC2476178

[9] Soulaphy C, Phouthone S, Khonesavanh P, Darouny P, Sonesavanh P, Khamphaphongphane B, et al Emergence of chikungunya in Moonlapamok and Khong Districts, Champassak Province, the Lao People’s Democratic Republic, May to September 2012. Western Pacific Surveillance and Response Journal. 2013;4: 46–50. 10.5365/WPSAR.2012.3.4.017 23908956 PMC3729106

[10] Somlor S, Vongpayloth K, Diancourt L, Buchy P, Duong V, Phonekeo D, et al Chikungunya virus emergence in the Lao PDR, 2012–2013. Ng LFP, editor. PLOS ONE. 2017;12: e0189879 10.1371/journal.pone.0189879 29284012 PMC5746231

[11] Khampapongpane B, Lewis HC, Ketmayoon P, Phonekeo D, Somoulay V, Khamsing A, et al National dengue surveillance in the Lao People’s Democratic Republic, 2006–2012: epidemiological and laboratory findings. Western Pacific Surveillance and Response Journal. 2014;5: 7–13. 10.5365/wpsar.2014.5.1.001 24734212 PMC3984965

[12] Erlanger TE, Keiser J, Utzinger J. Effect of dengue vector control interventions on entomological parameters in developing countries: a systematic review and meta-analysis. Medical and Veterinary Entomology. 2008;22: 203–221. 10.1111/j.1365-2915.2008.00740.x 18816269

[13] Nambanya S. Status of Dengue Fever/Dengue Haemorrhagic Fever in Lao People’s Democratic Republic. Dengue Bulletin. 1997;21: 115–116.

[14] Phommasak B. Dengue Haemmorrhagic fever control activities in Vientiane. Health and Social Welfare Services, Vientiane, Lao PDR; 1990.

[15] MNREPCD. Lao People’s Democratic Republic National Implementation Plan Under Stockholm Convention on Persistant Organic Pollutants. Ministry of Natural Resources and Environment Pollution Control Department. 147pp; 2016.

[16] Ranson H, Burhani J, Lumjuan N, Black IV WC. Insecticide resistance in dengue vectors. TropIKA net. 2010;1: 1–12.

[17] Moyes CL, Vontas J, Martins AJ, Ng LC, Koou SY, Dusfour I, et al Contemporary status of insecticide resistance in the major Aedes vectors of arboviruses infecting humans. Sinnis P, editor. PLOS Neglected Tropical Diseases. 2017;11: e0005625 10.1371/journal.pntd.0005625 28727779 PMC5518996

[18] Mouchet. La résistance aux insecticides des Aedes dans les régions d’Asie du Sud-Est et du Pacifique. Cah.ORSTOM, sér Ent méd, et Parasitol. 1972;X: 301–308.

[19] Jirakanjanakit N, Rongnoparut P, Saengtharatip S, Chareonviriyaphap T, Duchon S, Bellec C, et al Insecticide susceptible/resistance status in Aedes (Stegomyia) aegypti and Aedes (Stegomyia) albopictus (Diptera: Culicidae) in Thailand during 2003–2005. Journal of Economic Entomology. 2007;100: 545–550. 10.1603/0022-0493(2007)100[545:irsias]2.0.co;2 17461081

[20] Vontas J, Kioulos E, Pavlidi N, Morou E, della Torre A, Ranson H. Insecticide resistance in the major dengue vectors Aedes albopictus and Aedes aegypti. Pesticide Biochemistry and Physiology. 2012;104: 126–131. 10.1016/j.pestbp.2012.05.008

[21] Kawada H, Oo SZM, Thaung S, Kawashima E, Maung YNM, Thu HM, et al Co-occurrence of Point Mutations in the Voltage-Gated Sodium Channel of Pyrethroid-Resistant Aedes aegypti Populations in Myanmar. Kittayapong P, editor. PLoS Neglected Tropical Diseases. 2014;8: e3032 10.1371/journal.pntd.0003032 25077956 PMC4117438

[22] Li C-X, Kaufman PE, Xue R-D, Zhao M-H, Wang G, Yan T, et al Relationship between insecticide resistance and kdr mutations in the dengue vector Aedes aegypti in Southern China. Parasites & Vectors. 2015;8: 325 10.1186/s13071-015-0933-z 26068925 PMC4475621

[23] Boyer S, Lopes S, Prasetyo D, Hustedt J, Sarady AS, Doum D, et al Resistance of Aedes aegypti (Diptera: Culicidae) Populations to Deltamethrin, Permethrin, and Temephos in Cambodia. Asia Pacific Journal of Public Health. 2018;30: 158–166. 10.1177/1010539517753876 29502428

[24] Labbé P, Alout H, Djogbénou L, Pasteur N, Weill M. Evolution of Resistance to Insecticide in Disease Vectors Genetics and Evolution of Infectious Disease. Elsevier; 2011 pp. 363–409. 10.1016/B978-0-12-384890-1.00014-5

[25] Hemingway J, Hawkes NJ, McCarroll L, Ranson H. The molecular basis of insecticide resistance in mosquitoes. Insect Biochemistry and Molecular Biology. 2004;34: 653–665. 10.1016/j.ibmb.2004.03.018 15242706

[26] Brengues C, Hawkes NJ, Chandre F, Mccarroll L, Duchon S, Guillet P, et al Pyrethroid and DDT cross-resistance in Aedes aegypti is correlated with novel mutations in the voltage-gated sodium channel gene. Medical and Veterinary Entomology. 2003;17: 87–94. 10.1046/j.1365-2915.2003.00412.x 12680930

[27] Marcombe S, Carron A, Darriet F, Etienne M, Agnew P, Tolosa M, et al Reduced efficacy of pyrethroid space sprays for dengue control in an area of Martinique with pyrethroid resistance. The American journal of tropical medicine and hygiene. 2009;80: 745–751. 19407118

[28] Haddi K, Tomé HVV, Du Y, Valbon WR, Nomura Y, Martins GF, et al Detection of a new pyrethroid resistance mutation (V410L) in the sodium channel of Aedes aegypti: a potential challenge for mosquito control. Scientific Reports. 2017;7: 46549 10.1038/srep46549 28422157 PMC5396194

[29] Hirata K, Komagata O, Itokawa K, Yamamoto A, Tomita T, Kasai S. A Single Crossing-Over Event in Voltage-Sensitive Na+ Channel Genes May Cause Critical Failure of Dengue Mosquito Control by Insecticides. Dinglasan RR, editor. PLoS Neglected Tropical Diseases. 2014;8: e3085 10.1371/journal.pntd.0003085 25166902 PMC4148226

[30] Stenhouse SA, Plernsub S, Yanola J, Lumjuan N, Dantrakool A, Choochote W, et al Detection of the V1016G mutation in the voltage-gated sodium channel gene of Aedes aegypti (Diptera: Culicidae) by allele-specific PCR assay, and its distribution and effect on deltamethrin resistance in Thailand. Parasites & Vectors. 2013;6: 253 10.1186/1756-3305-6-253 24059267 PMC3765916

[31] Kawada H, Higa Y, Komagata O, Kasai S, Tomita T, Thi Yen N, et al Widespread Distribution of a Newly Found Point Mutation in Voltage-Gated Sodium Channel in Pyrethroid-Resistant Aedes aegypti Populations in Vietnam. Aksoy S, editor. PLoS Neglected Tropical Diseases. 2009;3: e527 10.1371/journal.pntd.0000527 19806205 PMC2754656

[32] Kasai S, Komagata O, Itokawa K, Shono T, Ng LC, Kobayashi M, et al Mechanisms of Pyrethroid Resistance in the Dengue Mosquito Vector, Aedes aegypti: Target Site Insensitivity, Penetration, and Metabolism. Kittayapong P, editor. PLoS Neglected Tropical Diseases. 2014;8: e2948 10.1371/journal.pntd.0002948 24945250 PMC4063723

[33] Plernsub S, Saingamsook J, Yanola J, Lumjuan N, Tippawangkosol P, Sukontason K, et al Additive effect of knockdown resistance mutations, S989P, V1016G and F1534C, in a heterozygous genotype conferring pyrethroid resistance in Aedes aegypti in Thailand. Parasites & Vectors. 2016;9 10.1186/s13071-016-1713-0 27460671 PMC4962480

[34] Hamid PH, Prastowo J, Widyasari A, Taubert A, Hermosilla C. Knockdown resistance (kdr) of the voltage-gated sodium channel gene of Aedes aegypti population in Denpasar, Bali, Indonesia. Parasites Vectors. 2017;10: 283 10.1186/s13071-017-2215-4 28583207 PMC5460344

[35] Al Nazawi AM, Aqili J, Alzahrani M, McCall PJ, Weetman D. Combined target site (kdr) mutations play a primary role in highly pyrethroid resistant phenotypes of Aedes aegypti from Saudi Arabia. Parasites & Vectors. 2017;10 10.1186/s13071-017-2096-6 28347352 PMC5368989

[36] Karunaratne S, Hemingway J. Malathion resistance and prevalence of the malathion carboxylesterase mechanism in populations of mosquito vectors of disease in Sri Lanka. Bulletin of the World Health Organization. 2001;79: 1060–1064. 11731814 PMC2566687

[37] Faucon F, Dusfour I, Gaude T, Navratil V, Boyer F, Chandre F, et al Identifying genomic changes associated with insecticide resistance in the dengue mosquito *Aedes aegypti* by deep targeted sequencing. Genome Research. 2015;25: 1347–1359. 10.1101/gr.189225.115 26206155 PMC4561493

[38] Poupardin R, Srisukontarat W, Yunta C, Ranson H. Identification of Carboxylesterase Genes Implicated in Temephos Resistance in the Dengue Vector Aedes aegypti. Benedict MQ, editor. PLoS Neglected Tropical Diseases. 2014;8: e2743 10.1371/journal.pntd.0002743 24651719 PMC3961196

[39] Marcombe S, Poupardin R, Darriet F, Reynaud S, Bonnet J, Strode C, et al Exploring the molecular basis of insecticide resistance in the dengue vector Aedes aegypti: a case study in Martinique Island (French West Indies). BMC Genomics. 2009;10: 494 10.1186/1471-2164-10-494 19857255 PMC2770535

[40] Pethuan S, Jirakanjanakit N, Saengtharatip S, Chareonviriyaphap T, Kaewpa D, Rongnoparut P, et al Biochemical studies of insecticide resistance in Aedes (Stegomyia) aegypti and Aedes (Stegomyia) albopictus (Diptera: Culicidae) in Thailand. Trop Biomed. 2007;24: 7–15. 17568372

[41] Somwang P, Yanola J, Suwan W, Walton C, Lumjuan N, Prapanthadara L, et al Enzymes-based resistant mechanism in pyrethroid resistant and susceptible Aedes aegypti strains from northern Thailand. Parasitology Research. 2011;109: 531–537. 10.1007/s00436-011-2280-0 21336645

[42] Marcombe S, Farajollahi A, Healy SP, Clark GG, Fonseca DM. Insecticide Resistance Status of United States Populations of Aedes albopictus and Mechanisms Involved. Adelman ZN, editor. PLoS ONE. 2014;9: e101992 10.1371/journal.pone.0101992 25013910 PMC4094391

[43] Chuaycharoensuk T, Juntarajumnong W, Boonyuan W, Bangs MJ, Akratanakul P, Thammapalo S, et al Frequency of pyrethroid resistance in Aedes aegypti and Aedes albopictus (Diptera: Culicidae) in Thailand. J Vector Ecol. 2011;36: 204–212. 10.1111/j.1948-7134.2011.00158.x 21635659

[44] WHO. Monitoring and managing insecticide resistance in Aedes mosquito populations. Interim guidance for entomologists. World Health Organization 2016 WHO/ZIKV/VC/16.1; 2016.

[45] WHO. Techniques to detect insecticide resistance mechanisms. World Health Organization, WHO_CDS_CPC_MAL_98.6.pdf; 1998.

[46] Brogdon WG, McAllister JC. Insecticide Resistance and Vector Control. Emerging Infectious Diseases. 1998;4: 9. 10.3201/eid0404.980410 PMC2640263 9866736

[47] Bradford M. Rapid and sensitive method for the quantitation of microgram quantities of protein utilizing the principle of protein-dye binding. Anal Biochem. 1976; 248–254. 10.1006/abio.1976.9999 942051

[48] Chen M, Du Y, Wu S, Nomura Y, Zhu G, Zhorov BS, et al Molecular evidence of sequential evolution of DDT- and pyrethroid-resistant sodium channel in Aedes aegypti. Lenhart A, editor. PLoS Negl Trop Dis. 2019;13: e0007432 10.1371/journal.pntd.0007432 31158225 PMC6564045

[49] Harris AF, Rajatileka S, Ranson H. Pyrethroid Resistance in Aedes aegypti from Grand Cayman. The American Journal of Tropical Medicine and Hygiene. 2010;83: 277–284. 10.4269/ajtmh.2010.09-0623 20682868 PMC2911171

[50] Linss JG, Brito L, Garcia G, Araki A, Bruno R, Lima JB, et al Distribution and dissemination of the Val1016Ile and Phe1534Cys Kdr mutations in Aedes aegypti Brazilian natural populations. Parasit Vectors. 2014;7: 25 10.1186/1756-3305-7-25 24428880 PMC3912884

[51] Saavedra-Rodriguez K, Urdaneta-Marquez L, Rajatileka S, Moulton M, Flores AE, Fernandez I, et al A mutation in the voltage-gated sodium channel gene associated with pyrethroid resistance in Latin American. Insect Molecular Biology. 2007;16: 785–798. 10.1111/j.1365-2583.2007.00774.x 18093007

[52] Yanola J, Somboon P, Walton C, Nachaiwieng W, Somwang P, Prapanthadara L. High-throughput assays for detection of the F1534C mutation in the voltage-gated sodium channel gene in permethrin-resistant Aedes aegypti and the distribution of this mutation throughout Thailand: High-throughput assays to detect the F1534C mutation in sodium channel gene of the Aedes aegypti. Tropical Medicine & International Health. 2011;16: 501–509. 10.1111/j.1365-3156.2011.02725.x 21342372

[53] Faucon F, Gaude T, Dusfour I, Navratil V, Corbel V, Juntarajumnong W, et al In the hunt for genomic markers of metabolic resistance to pyrethroids in the mosquito Aedes aegypti: An integrated next-generation sequencing approach. Reiner RC, editor. PLOS Neglected Tropical Diseases. 2017;11: e0005526 10.1371/journal.pntd.0005526 28379969 PMC5393893

[54] Pfaffl MW. A new mathematical model for relative quantification in real-time RT-PCR. Nucleic Acids Research. 2001;29: 45e–445. 10.1093/nar/29.9.e45 11328886 PMC55695

[55] Abbott W. A method of computing the effectiveness of an insecticide. J Econ Entomol. 1925;18: 265–267.

[56] Raymond M, Rousset F. GENEPOP (Version 1.2): Population Genetics Software for Exact Tests and Ecumenicism. Journal of Heredity. 1995;86: 248–249. 10.1093/oxfordjournals.jhered.a111573

[57] Rousset F. genepop’007: a complete re-implementation of the genepop software for Windows and Linux. Molecular Ecology Resources. 2008;8: 103–106. 10.1111/j.1471-8286.2007.01931.x 21585727

[58] Tangena J-AA, Marcombe S, Thammavong P, Chonephetsarath S, Somphong B, Sayteng K, et al Bionomics and insecticide resistance of the arboviral vector Aedes albopictus in northern Lao PDR. Moreira LA, editor. PLOS ONE. 2018;13: e0206387 10.1371/journal.pone.0206387 30359425 PMC6201963

[59] Marcombe S, Chonephetsarath S, Thammavong P, Brey PT. Alternative insecticides for larval control of the dengue vector Aedes aegypti in Lao PDR: insecticide resistance and semi-field trial study. Parasites & Vectors. 2018;11: 616 10.1186/s13071-018-3187-8 30509299 PMC6278129

[60] Goindin D, Delannay C, Gelasse A, Ramdini C, Gaude T, Faucon F, et al Levels of insecticide resistance to deltamethrin, malathion, and temephos, and associated mechanisms in Aedes aegypti mosquitoes from the Guadeloupe and Saint Martin islands (French West Indies). Infectious Diseases of Poverty. 2017;6: 38 10.1186/s40249-017-0254-x 28187780 PMC5303256

[61] Flores-Suarez AE, Ponce-Garcia G, Lopez-Monroy B, Villanueva-Segura OK, Rodriguez-Sanchez IP, Arredondo-Jimenez JI, et al Current Status of the Insecticide Resistance in Aedes aegypti (Diptera: Culicidae) from Mexico In: Trdan S, editor. Insecticides Resistance. InTech; 2016 10.5772/61526

[62] Viana-Medeiros PF, Bellinato DF, Martins AJ, Valle D. Insecticide resistance, associated mechanisms and fitness aspects in two Brazilian Stegomyia aegypti (= Aedes aegypti) populations: Insecticide resistance in Brazilian S. aegypti. Medical and Veterinary Entomology. 2017;31: 340–350. 10.1111/mve.12241 28752548

[63] Marcombe S, Bobichon J, Somphong B, Phommavan N, Maithaviphet S, Nambanya S, et al Insecticide resistance status of malaria vectors in Lao PDR. Hansen IA, editor. PLOS ONE. 2017;12: e0175984 10.1371/journal.pone.0175984 28437449 PMC5402946

[64] Ishak IH, Riveron JM, Ibrahim SS, Stott R, Longbottom J, Irving H, et al The Cytochrome P450 gene CYP6P12 confers pyrethroid resistance in kdr-free Malaysian populations of the dengue vector Aedes albopictus. Scientific Reports. 2016;6 10.1038/s41598-016-0015-2 27094778 PMC4837359

[65] Rajatileka S, Black WC, Saavedra-Rodriguez K, Trongtokit Y, Apiwathnasorn C, McCall PJ, et al Development and application of a simple colorimetric assay reveals widespread distribution of sodium channel mutations in Thai populations of Aedes aegypti. Acta Tropica. 2008;108: 54–57. 10.1016/j.actatropica.2008.08.004 18801327

[66] Wuliandari J, Lee S, White V, Tantowijoyo W, Hoffmann A, Endersby-Harshman N. Association between Three Mutations, F1565C, V1023G and S996P, in the Voltage-Sensitive Sodium Channel Gene and Knockdown Resistance in Aedes aegypti from Yogyakarta, Indonesia. Insects. 2015;6: 658–685. 10.3390/insects6030658 26463408 PMC4598657

[67] Fernando SD, Hapugoda M, Perera R, Saavedra-Rodriguez K, Black WC, De Silva NK. First report of V1016G and S989P knockdown resistant (kdr) mutations in pyrethroid-resistant Sri Lankan Aedes aegypti mosquitoes. Parasites & Vectors. 2018;11: 526 10.1186/s13071-018-3113-0 30257701 PMC6158842

[68] Brooke BD. kdr: can a single mutation produce an entire insecticide resistance phenotype? Transactions of the Royal Society of Tropical Medicine and Hygiene. 2008;102: 524–525. 10.1016/j.trstmh.2008.01.001 18295809

[69] Marcombe S, Mathieu RB, Pocquet N, Riaz M-A, Poupardin R, Sélior S, et al Insecticide Resistance in the Dengue Vector Aedes aegypti from Martinique: Distribution, Mechanisms and Relations with Environmental Factors. Hansen IA, editor. PLoS ONE. 2012;7: e30989 10.1371/journal.pone.0030989 22363529 PMC3283601

[70] Polson KA, Brogdon WG, Rawlins SC, Chadee DD. Characterization of insecticide resistance in Trinidadian strains of Aedes aegypti mosquitoes. Acta Tropica. 2011;117: 31–38. 10.1016/j.actatropica.2010.09.005 20858454

[71] Stevenson BJ, Pignatelli P, Nikou D, Paine MJI. Pinpointing P450s Associated with Pyrethroid Metabolism in the Dengue Vector, Aedes aegypti: Developing New Tools to Combat Insecticide Resistance. Kent Crockett RJ, editor. PLoS Neglected Tropical Diseases. 2012;6: e1595 10.1371/journal.pntd.0001595 22479665 PMC3313934

[72] Grigoraki L, Lagnel J, Kioulos I, Kampouraki A, Morou E, Labbé P, et al Transcriptome Profiling and Genetic Study Reveal Amplified Carboxylesterase Genes Implicated in Temephos Resistance, in the Asian Tiger Mosquito Aedes albopictus. McCall PJ, editor. PLOS Neglected Tropical Diseases. 2015;9: e0003771 10.1371/journal.pntd.0003771 26000638 PMC4441504

[73] Grigoraki L, Balabanidou V, Meristoudis C, Miridakis A, Ranson H, Swevers L, et al Functional and immunohistochemical characterization of CCEae3a, a carboxylesterase associated with temephos resistance in the major arbovirus vectors Aedes aegypti and Ae. albopictus. Insect Biochemistry and Molecular Biology. 2016;74: 61–67. 10.1016/j.ibmb.2016.05.007 27180726

[74] Weill M, Lutfalla G, Mogensen K, Chandre F, Berthomieu A, Berticat C, et al Insecticide resistance in mosquito vectors. Nature. 2003;423: 136–137. 10.1038/423136b 12736674

