# Supplementary material for: Distribution of insecticide resistance and mechanisms involved in the arbovirus vector Aedes aegypti in Laos and implication for vector control
Source: PLoS Negl Trop Dis. 2019 Dec 12;13(12):e0007852. doi: 10.1371/journal.pntd.0007852 (PMC6932826; doi:10.1371/journal.pntd.0007852)
Supplement: S1 Table — (PDF) [file pntd.0007852.s001.pdf]

Table S1. Primers used for kdr screening and CNV detection

accession number	primer name	gene target	fragment (bp)	sequence 5' - 3'	hybridation temperature(°C)
AAEL006019	Gly1016f	V1016G kdr mutation on VGSC gene		ACCGACAAATT GTTTCCC	60
	Val1016r		60	GCGGGCAGCAAGGCTAAGAAAAGGTTAATTA	60
	Gly1016r		80	GCGGGCAGGGCGGCGGGGGCGGGGCCAGCAAGG CTAAGAAAAGGTTAACTC	60
EU259811	CP –r	F1534C kdr mutation on VGSC gene		TCTGCTCGTTGAAGTTGTCGAT	60
	F1534f		93	GCGGGCTCTACTTTGTGTTCTTCATCATATT	60
	C1534f		113	GCGGGCAGGGCGGCGGGGGCGGGGCCTCTACTTT GTGTTCTTCATCATGTG	60
AAEL003844		CCEAE3A	130	TCTAAGAAACCCGAATATGACG<br>TTGAGGAGGCACGAACAG	54
AAEL014617		CYP9J28	197	CTATTTCGGAGTCCTAGTGGCC<br>CTTTGACTCCTCGGTACTTGTCG	60
AAEL014614		CYP9Jlike	121	GGAAGCGTTGAGCATGTGTG<br>AACTGTGAAACCGTGGGGTC	60
AAEL014893		CYP6BB2	143	AGTTCAAGGGCCGAGGATTG<br>CGGATCCACGAAAATTCCGC	60
AAEL014891		CYP6P12	152	TTCACCTTCAGCGAAGACCC<br>GATTAGGTGCGGCGTCCTTA	60
AAEL007808		CYP4D39	132	AGTCCTGGAAGTTCTGCACG<br>AAGGCGACTTTCCGACGAAT	60
