# Supplementary material for: Distribution of insecticide resistance and mechanisms involved in the arbovirus vector Aedes aegypti in Laos and implication for vector control
Source: PLoS Negl Trop Dis. 2019 Dec 12;13(12):e0007852. doi: 10.1371/journal.pntd.0007852 (PMC6932826; doi:10.1371/journal.pntd.0007852)
Supplement: S2 Table — (PDF) [file pntd.0007852.s002.pdf]

Table S2: Genes targeted for CNV analysis

Accession number	Gene name	Description	Previous involvement in resistance	References
AAEL023844	CCEAE3A	Carboxy/choline Esterase	temephos	(Grigoraki et al. 2016)
AAEL014617	CYP9J28	Cytochrome P450	pyrethroids	(Stevenson et al. 2012)
AAEL014614	CYP9J-like	Cytochrome P450	pyrethroids	(Stevenson et al. 2012)
AAEL014893	CYP6BB2	Cytochrome P450	deltamethrin, permethrin	(Kasai et al. 2014; Faucon et al. 2017)
AAEL014891	CYP6P12	Cytochrome P450	permethrin/deltamethrin (Ae. albopictus)	(Ishak et al 2016)
AAEL007808	CYP4D39	Cytochrome P450	control genes (no CNV variation across various continents)	(Faucon et al. 2015, 2017)
AAEL005950	AAEL005950	Chloride channel protein 2	control genes (no CNV variation across various continents)	(Faucon et al. 2015, 2017)
