# Supplementary material for: Distribution of insecticide resistance and mechanisms involved in the arbovirus vector Aedes aegypti in Laos and implication for vector control
Source: PLoS Negl Trop Dis. 2019 Dec 12;13(12):e0007852. doi: 10.1371/journal.pntd.0007852 (PMC6932826; doi:10.1371/journal.pntd.0007852)
Supplement: S3 Table — (PDF) [file pntd.0007852.s003.pdf]

Supplementary table 3. Crude data from GENEPOP

1. HARDY WEINDBERG EXPECTATIONS

Fri Sep 18 16:48:56 AWST 2015

```
Genepop  version 4.2: Hardy-Weinberg test

File: 164856 (mosquito populations in Laos)

Number of populations detected:    11
Number of loci detected:           2


Estimation of exact P-Values by the Markov chain method.
---------------------------------------------
Markov chain parameters for all tests:
Dememorization:              1000
Batches:                     100
Iterations per batch:        1000
Hardy Weinberg: Probability test
        ************************
==========================================
All locus, all populations
==========================================
All (Fisher's method) :
 Chi2 :    Infinity
 Df   :    38.0000
 Prob :    High. sign.

==========================================
     Results by locus
==========================================

Locus "V1016G"
-----------------------------------------
                              Fis estimates
```

POPULATIONS	P-value	S.E.	W&C	R&H	Steps
Kao-gnot I	1	0	-0.0266	-0.0267	70555 switches
Dongpalab	0.8201	0.0017	-0.0311	-0.0312	81545 switches
Oudomphon	0.5004	0.0038	0.0742	0.0746	82352 switches
Phailom	1	0	-0.0374	-0.0376	14004 switches
Taling	1	0	-0.0353	-0.0354	63121 switches
Khomkhuang	0.0043	0.0004	0.4843	0.4877	12388 switches
Thongchale	0	0	0.823	0.8302	75862 switches
Thatnoy	1	0	-0.0093	-0.0094	69362 switches
Lakhonphen	0.3863	0.0022	0.0772	0.0776	38455 switches
Khonsaiy	-				
Saisa-art	-				

```
All (Fisher's method):
 Chi2:    Infinity
 Df   :    18.0000
 Prob :    High. sign.
```

Locus "F1534C"

Fis estimates

OPULATIONS	P-value	S.E.	W&C	R&H	Steps
**Kao-gnot I**	1	0	-0.0357	-0.0358	71687 switches
**Dongpalab**	0.8184	0.0018	-0.0379	-0.0381	82153 switches
**Oudomphon**	0.5054	0.0039	0.0742	0.0746	82189 switches
**Phailom**	1	0	-0.0543	-0.0546	25726 switches
**Taling**	1	0	-0.0597	-0.06	69106 switches
**Khomkhuang**	0.1575	0.0018	0.1929	0.194	16034 switches
**Thongchale**	0.5108	0.0027	-0.0877	-0.088	73975 switches
**Thatnoy**	1	0	-0.0372	-0.0374	71483 switches
**Lakhonphen**	1	0	-0.0556	-0.0558	30451 switches
**Khonsaiy**	0.1996	0.0026	-0.2115	-0.2132	75078 switches
**Saisa-art**	0.5751	0.0012	-0.1494	-0.1507	57405 switches

All (Fisher's method):
 Chi2:   11.1352
 Df   :   22.0000
 Prob :   0.9728

==

Results by population

==

Pop : Kao-gnot IPL

Fis estimates

----

locus	P-val	S.E.	W&C	R&H	Steps	
V1016G	0.8201	0.0017	-0.0311	-0.0312	81545	switches
F1534C	0.8184	0.0018	-0.0379	-0.0381	82153	switches

All (Fisher's method):
 Chi2 :   0.0000
 Df   :   4.0000
 Prob :   1

Pop : Dongpalab

Fis estimates

----

locus	P-val	S.E.	W&C	R&H	Steps	
V1016G	0.8201	0.0017	-0.0311	-0.0312	81545	switches
F1534C	0.8184	0.0018	-0.0379	-0.0381	82153	switches

All (Fisher's method):
 Chi2 :   0.7975
 Df   :   4.0000
 Prob :   0.9388

Pop : Oudomphon

Fis estimates

```
                              ---------------
locus        P-val   S.E.    W&C     R&H     Steps
----------- ------- ------- ------- ------- ------
V1016G       0.5004  0.0038   0.0742  0.0746  82352 switches
F1534C       0.5054  0.0039   0.0742  0.0746  82189 switches

All (Fisher's method):
 Chi2 :    2.7495
 Df   :    4.0000
 Prob :    0.6006

 Pop : Phailom
-----------------------------------------
                               Fis estimates
                              ---------------
locus        P-val   S.E.    W&C     R&H     Steps
----------- ------- ------- ------- ------- ------
V1016G       1.0000  0.0000  -0.0374 -0.0376  14004 switches
F1534C       1.0000  0.0000  -0.0543 -0.0546  25726 switches

All (Fisher's method):
 Chi2 :    0.0000
 Df   :    4.0000
 Prob :    1

 Pop : Taling
-----------------------------------------
                               Fis estimates
                              ---------------
locus        P-val   S.E.    W&C     R&H     Steps
----------- ------- ------- ------- ------- ------
V1016G       1.0000  0.0000  -0.0353 -0.0354  63121 switches
F1534C       1.0000  0.0000  -0.0597 -0.0600  69106 switches

All (Fisher's method):
 Chi2 :    0.0000
 Df   :    4.0000
 Prob :    1

 Pop : Khomkhuang
-----------------------------------------
                               Fis estimates
                              ---------------
locus        P-val   S.E.    W&C     R&H     Steps
----------- ------- ------- ------- ------- ------
V1016G       0.0043  0.0004   0.4843  0.4877  12388 switches
F1534C       0.1575  0.0018   0.1929  0.1940  16034 switches

All (Fisher's method):
 Chi2 :    14.6045
 Df   :    4.0000
 Prob :    0.0056

 Pop : Thongchaleun
-----------------------------------------
                               Fis estimates
                              ---------------
locus        P-val   S.E.    W&C     R&H     Steps
----------- ------- ------- ------- ------- ------
```

```
V1016G      0.0000  0.0000   0.8230  0.8302  75862 switches
F1534C      0.5108  0.0027  -0.0877 -0.0880  73975 switches

All (Fisher's method):
 Chi2 :    Infinity
 Df   :    4.0000
 Prob :    High. sign.

 Pop : Thatnoy
-----------------------------------------
                             Fis estimates
                            ---------------
locus       P-val   S.E.    W&C     R&H     Steps
----------- ------- ------- ------- ------- ------
V1016G      1.0000  0.0000  -0.0093 -0.0094  69362 switches
F1534C      1.0000  0.0000  -0.0372 -0.0374  71483 switches

All (Fisher's method):
 Chi2 :    0.0000
 Df   :    4.0000
 Prob :    1

 Pop : Lakhonpheng
-----------------------------------------
                             Fis estimates
                            ---------------
locus       P-val   S.E.    W&C     R&H     Steps
----------- ------- ------- ------- ------- ------
V1016G      0.3863  0.0022   0.0772  0.0776  38455 switches
F1534C      1.0000  0.0000  -0.0556 -0.0558  30451 switches

All (Fisher's method):
 Chi2 :    1.9024
 Df   :    4.0000
 Prob :    0.7537

 Pop : Khonsaiy
-----------------------------------------
                             Fis estimates
                            ---------------
locus       P-val   S.E.    W&C     R&H     Steps
----------- ------- ------- ------- ------- ------
V1016G       No information.
F1534C      0.1996  0.0026  -0.2115 -0.2132  75078 switches

 Pop : Saisa-art
-----------------------------------------
                             Fis estimates
                            ---------------
locus       P-val   S.E.    W&C     R&H     Steps
----------- ------- ------- ------- ------- ------
V1016G       No information.
F1534C      0.5751  0.0012  -0.1494 -0.1507  57405 switches
==========================================
Normal ending
```

2. KDR ALLELICS FREQUENCY

Tue Sep 29 16:11:12 AWST 2015

Genepop version 4.2, Genic differentiation for each population pair (Fisher's exact Probability test)

File: 161112 (mosquito populations in Laos)

Number of populations detected : 11
Number of loci detected : 2

Markov chain parameters
Dememorisation : 1000
Batches : 100
Iterations per batch : 1000

Locus: V1016G
=================================

Pop Alleles:

	1	2	Total
Kao-gnot	202	34	236
Dongpalab	108	60	168
Oudomphon	149	67	216
Phailom	188	8	196
Taling	206	26	232
Khomkhuan	208	8	216
Thongchal	168	42	210
Thatnoy	202	32	234
Lakhonphe	215	15	230
Khonsaiy	100	0	100
Saisa-art	102	0	102
Total:	1848	292	2140

P-value = 0 S.E. = 0 (88165 switches)

Locus	Population pair	P-Value	S.E.	Switches
V1016G	Dongpalab & Kao-gnot	0	0	90583
V1016G	Oudomphon & Kao-gnot	0.0001	0.0001	90808
V1016G	Oudomphon & Dongpalab	0.38695	0.00844	91192
V1016G	Phailom & Kao-gnot	0.00037	0.00018	86807
V1016G	Phailom & Dongpalab	0	0	89156
V1016G	Phailom & Oudomphon	0	0	89630
V1016G	Taling & Kao-gnot	0.32371	0.006	89328
V1016G	Taling & Dongpalab	0	0	90103
V1016G	Taling & Oudomphon	0	0	90805
V1016G	Taling & Phailom	0.00764	0.00101	85819
V1016G	Khomkhuan & Kao-gnot	0.00012	0.00008	87011
V1016G	Khomkhuan & Dongpalab	0	0	89298
V1016G	Khomkhuan & Oudomphon	0	0	90072
V1016G	Khomkhuan & Phailom	1	0	80202
V1016G	Khomkhuan & Taling	0.00437	0.00064	85571
V1016G	Thongchal & Kao-gnot	0.13356	0.00635	89949
V1016G	Thongchal & Dongpalab	0.0007	0.00042	90932
V1016G	Thongchal & Oudomphon	0.01155	0.00176	91040
V1016G	Thongchal & Phailom	0	0	87747
V1016G	Thongchal & Taling	0.01064	0.00138	89795
V1016G	Thongchal & Khomkhuan	0	0	88306
V1016G	Thatnoy & Kao-gnot	0.89038	0.00209	89673
V1016G	Thatnoy & Dongpalab	0	0	90597
V1016G	Thatnoy & Oudomphon	0.00004	0.00004	91019
V1016G	Thatnoy & Phailom	0.00131	0.00044	86744
V1016G	Thatnoy & Taling	0.48435	0.0058	88753
V1016G	Thatnoy & Khomkhuan	0.00022	0.00011	86609
V1016G	Thatnoy & Thongchal	0.10458	0.00549	89533
V1016G	Lakhonphe & Kao-gnot	0.00705	0.00087	87993
V1016G	Lakhonphe & Dongpalab	0	0	89748
V1016G	Lakhonphe & Oudomphon	0	0	90386
V1016G	Lakhonphe & Phailom	0.29191	0.00428	83224
V1016G	Lakhonphe & Taling	0.09357	0.0035	87378
V1016G	Lakhonphe & Khomkhuan	0.1996	0.00353	83067
V1016G	Lakhonphe & Thongchal	0.00005	0.00005	88649
V1016G	Lakhonphe & Thatnoy	0.0134	0.00132	87643
V1016G	Khonsaiy & Kao-gnot	0	0	84246
V1016G	Khonsaiy & Dongpalab	0	0	88204
V1016G	Khonsaiy & Oudomphon	0	0	88109
V1016G	Khonsaiy & Phailom	0.05625	0.00159	72037
V1016G	Khonsaiy & Taling	0.00006	0.00003	82277
V1016G	Khonsaiy & Khomkhuan	0.05807	0.00145	71365
V1016G	Khonsaiy & Thongchal	0	0	86035
V1016G	Khonsaiy & Thatnoy	0	0	84010
V1016G	Khonsaiy & Lakhonphe	0.00733	0.00062	77836
V1016G	Saisa-art & Kao-gnot	0	0	84317
V1016G	Saisa-art & Dongpalab	0	0	88270
V1016G	Saisa-art & Oudomphon	0	0	88404
V1016G	Saisa-art & Phailom	0.0535	0.00142	71940
V1016G	Saisa-art & Taling	0.00003	0.00002	82586
V1016G	Saisa-art & Khomkhuan	0.05778	0.00141	71452
V1016G	Saisa-art & Thongchal	0	0	86174
V1016G	Saisa-art & Thatnoy	0	0	84467
V1016G	Saisa-art & Lakhonphe	0.00608	0.00049	77988
V1016G	Saisa-art & Khonsaiy	No table		

Locus: F1534C

==================================

Pop Alleles:

Pop	1	2	Total
Kao-gnot	35	201	236
Dongpalab	67	107	174
Oudomphon	67	149	216
Phailom	11	185	196
Taling	29	203	232
Khomkhuan	9	207	216
Thongchal	38	172	210
Thatnoy	35	199	234
Lakhonphe	13	217	230
Khonsaiy	33	67	100
Saisa-art	88	14	102
Total:	425	1721	2146

P-value = 0 S.E. = 0 (89618 switches)

Locus	Population pair	P-Value	S.E.	Switches
F1534C	Dongpalab & Kao-gnot	0	0	90934
F1534C	Oudomphon & Kao-gnot	0.00007	0.00007	90853
F1534C	Oudomphon & Dongpalab	0.12939	0.00642	91564
F1534C	Phailom & Kao-gnot	0.0019	0.00054	87813
F1534C	Phailom & Dongpalab	0	0	90162
F1534C	Phailom & Oudomphon	0	0	90070
F1534C	Taling & Kao-gnot	0.50459	0.00573	89385
F1534C	Taling & Dongpalab	0	0	90644
F1534C	Taling & Oudomphon	0	0	90615
F1534C	Taling & Phailom	0.01946	0.0016	86651
F1534C	Khomkhuan & Kao-gnot	0.00022	0.00011	87223
F1534C	Khomkhuan & Dongpalab	0	0	89598
F1534C	Khomkhuan & Oudomphon	0	0	89916
F1534C	Khomkhuan & Phailom	0.50851	0.00432	82106
F1534C	Khomkhuan & Taling	0.00171	0.00039	86239
F1534C	Thongchal & Kao-gnot	0.37754	0.00739	89787
F1534C	Thongchal & Dongpalab	0	0	91046
F1534C	Thongchal & Oudomphon	0.00255	0.00057	90866
F1534C	Thongchal & Phailom	0.00009	0.00006	87748
F1534C	Thongchal & Taling	0.1083	0.00496	89578
F1534C	Thongchal & Khomkhuan	0	0	87851
F1534C	Thatnoy & Kao-gnot	1	0	89730
F1534C	Thatnoy & Dongpalab	0	0	90909
F1534C	Thatnoy & Oudomphon	0.00005	0.00005	91007
F1534C	Thatnoy & Phailom	0.0017	0.00035	87667
F1534C	Thatnoy & Taling	0.49113	0.00678	89366
F1534C	Thatnoy & Khomkhuan	0.00005	0.00004	87352
F1534C	Thatnoy & Thongchal	0.4463	0.0072	89768

F1534C	Lakhonphe & Kao-gnot	0.00083	0.00022	88002
F1534C	Lakhonphe & Dongpalab	0	0	90061
F1534C	Lakhonphe & Oudomphon	0	0	90199
F1534C	Lakhonphe & Phailom	1	0	83481
F1534C	Lakhonphe & Taling	0.01473	0.00166	87235
F1534C	Lakhonphe & Khomkhuan	0.51619	0.00403	82942
F1534C	Lakhonphe & Thongchal	0.00005	0.00005	88365
F1534C	Lakhonphe & Thatnoy	0.00083	0.00025	87894
F1534C	Khonsaiy & Kao-gnot	0.00025	0.00019	88123
F1534C	Khonsaiy & Dongpalab	0.43125	0.00669	89638
F1534C	Khonsaiy & Oudomphon	0.79388	0.00339	89897
F1534C	Khonsaiy & Phailom	0	0	86081
F1534C	Khonsaiy & Taling	0.00007	0.00007	87924
F1534C	Khonsaiy & Khomkhuan	0	0	85825
F1534C	Khonsaiy & Thongchal	0.00445	0.00072	88627
F1534C	Khonsaiy & Thatnoy	0.0004	0.00023	88030
F1534C	Khonsaiy & Lakhonphe	0	0	86383
F1534C	Saisa-art & Kao-gnot	0	0	90446
F1534C	Saisa-art & Dongpalab	0	0	90070
F1534C	Saisa-art & Oudomphon	0	0	90347
F1534C	Saisa-art & Phailom	0	0	89684
F1534C	Saisa-art & Taling	0	0	90246
F1534C	Saisa-art & Khomkhuan	0	0	89430
F1534C	Saisa-art & Thongchal	0	0	90088
F1534C	Saisa-art & Thatnoy	0	0	90025
F1534C	Saisa-art & Lakhonphe	0	0	89800
F1534C	Saisa-art & Khonsaiy	0	0	88702

==================================

P-value for each population pair across all loci
(Fisher's method)

Population pair	Chi2	df	P-Value
**Kao-gnot IPL & Dongpalab**	Infinity	4	Highly sign.
**Kao-gnot IPL & Oudomphon**	37.55471	4	0
**Dongpalab & Oudomphon**	5.988768	4	0.199989
**Kao-gnot IPL & Phailom**	28.33582	4	0.000011
**Dongpalab & Phailom**	Infinity	4	Highly sign.
**Oudomphon & Phailom**	Infinity	4	Highly sign.
**Kao-gnot IPL & Taling**	3.623833	4	0.459301
**Dongpalab & Taling**	Infinity	4	Highly sign.
**Oudomphon & Taling**	Infinity	4	Highly sign.
**Phailom & Taling**	17.6275	4	0.001459
**Kao-gnot IPL & Khomkhuang**	34.8998	4	0
**Dongpalab & Khomkhuang**	Infinity	4	Highly sign.
**Oudomphon & Khomkhuang**	Infinity	4	Highly sign.
**Phailom & Khomkhuang**	1.352541	4	0.8524
**Taling & Khomkhuang**	23.60851	4	0.000096
**Kao-gnot IPL & Thongchaleun**	5.974566	4	0.201056
**Dongpalab & Thongchaleun**	Infinity	4	Highly sign.
**Oudomphon & Thongchaleun**	20.86546	4	0.000337
**Phailom & Thongchaleun**	Infinity	4	Highly sign.
**Taling & Thongchaleun**	13.53197	4	0.008949

**Khomkhuang & Thongchaleun**	Infinity	4	Highly sign.
**Kao-gnot IPL & Thatnoy**	0.232214	4	0.993759
**Dongpalab & Thatnoy**	Infinity	4	Highly sign.
**Oudomphon & Thatnoy**	40.06024	4	0
**Phailom & Thatnoy**	26.02971	4	0.000031
**Taling & Thatnoy**	2.871988	4	0.579471
**Khomkhuang & Thatnoy**	36.65074	4	0
**Thongchaleun & Thatnoy**	6.129134	4	0.18971
**Kao-gnot IPL & Lakhonpheng**	24.09763	4	0.000076
**Dongpalab & Lakhonpheng**	Infinity	4	Highly sign.
**Oudomphon & Lakhonpheng**	Infinity	4	Highly sign.
**Phailom & Lakhonpheng**	2.462619	4	0.651342
**Taling & Lakhonpheng**	13.17383	4	0.010457
**Khomkhuang & Lakhonpheng**	4.545441	4	0.337193
**Thongchaleun & Lakhonpheng**	39.61395	4	0
**Thatnoy & Lakhonpheng**	22.81317	4	0.000138
**Kao-gnot IPL & Khonsaiy**	Infinity	4	Highly sign.
**Dongpalab & Khonsaiy**	Infinity	4	Highly sign.
**Oudomphon & Khonsaiy**	Infinity	4	Highly sign.
**Phailom & Khonsaiy**	Infinity	4	Highly sign.
**Taling & Khonsaiy**	38.57636	4	0
**Khomkhuang & Khonsaiy**	Infinity	4	Highly sign.
**Thongchaleun & Khonsaiy**	Infinity	4	Highly sign.
**Thatnoy & Khonsaiy**	Infinity	4	Highly sign.
**Lakhonpheng & Khonsaiy**	Infinity	4	Highly sign.
**Kao-gnot IPL & Saisa-art**	Infinity	4	Highly sign.
**Dongpalab & Saisa-art**	Infinity	4	Highly sign.
**Oudomphon & Saisa-art**	Infinity	4	Highly sign.
**Phailom & Saisa-art**	Infinity	4	Highly sign.
**Taling & Saisa-art**	Infinity	4	Highly sign.
**Khomkhuang & Saisa-art**	Infinity	4	Highly sign.
**Thongchaleun & Saisa-art**	Infinity	4	Highly sign.
**Thatnoy & Saisa-art**	Infinity	4	Highly sign.
**Lakhonpheng & Saisa-art**	Infinity	4	Highly sign.
**Khonsaiy & Saisa-art**	Infinity	2	Highly sign.

```
=================================

P-value across all loci
(Fisher's method)
-----------------------------------------------------
Locus          P-Value
-------------  --------
V1016G         0
F1534C         0

All: Chi2= Infinity (df= 4), highly significant

Normal ending.
```

3. GENOTYPIQUE LINKAGE DISEQUILIBRIUM

Fri Oct 9 10:34:54 AWST 2015

```
Genepop version 4.2, Genotypic linkage disequilibrium

File: 103454 (mosquito populations in Laos)

Number of populations detected : 11
Number of loci detected        : 2

Markov chain parameters
        Dememorisation       : 1000
        Batches              : 100
        Iterations per batch : 1000
```

Pop	Locus#1	Locus#2	P-Value	S.E.	Switches
**Kao-gnot IPL**	V1016G	F1534C	0	0	30336
**Dongpalab**	V1016G	F1534C	0	0	64655
**Oudomphon**	V1016G	F1534C	0	0	68145
**Phailom**	V1016G	F1534C	0	0	59207
**Taling**	V1016G	F1534C	0	0	19150
**Khomkhuang**	V1016G	F1534C	0	0	6726
**Thongchaleun**	V1016G	F1534C	0	0	34863
**Thatnoy**	V1016G	F1534C	0	0	29633
**Lakhonpheng**	V1016G	F1534C	0	0	29033
**Khonsaiy**	V1016G	F1534C	No contingency table		
**Saisa-art**	V1016G	F1534C	No contingency table		

```
P-value for each locus pair across all populations
(Fisher's method)
-----------------------------------------------------
Locus pair                    Chi2      df   P-Value
--------------------          --------  ---  --------
V1016G        & F1534C         Infinity 18   Highly sign.

Normal ending.
```
