# Supplementary material for: Distribution of insecticide resistance and mechanisms involved in the arbovirus vector Aedes aegypti in Laos and implication for vector control
Source: PLoS Negl Trop Dis. 2019 Dec 12;13(12):e0007852. doi: 10.1371/journal.pntd.0007852 (PMC6932826; doi:10.1371/journal.pntd.0007852)
Supplement: S4 Table — (PDF) [file pntd.0007852.s004.pdf]

Supplementary Table 4. V1016G and F1534C genotypic differentiation and haplotype differentiation between dead and live mosquitoes for DDT and permethrin

**V1016G**				**F1534C**			
	**DDT**				**DDT**		
	SS	RS	RR		SS	RS	RR
Alive	27	30	3	Alive	3	30	27
Dead	16	13	6	Dead	5	14	16
p-value	*0.42*			*p-value*	*0.62*		
	**Permethrin**				**Permethrin**		
	SS	RS	RR		SS	RS	RR
Alive	23	25	8	Alive	11	26	22
Dead	21	15	5	Dead	5	16	20
p-value	*0.47*			*p-value*	*0.27*		

DDT survival

Haplotypes V1016G/F1534C	ALIVE	DEAD
SS/RR	**27**	**16**
RS/RS	**30**	**13**
RR/SS	**3**	**5**
RR/RS	**0**	**1**
chisquare test pvalue *0.19*		

Permethrin survival

Haplotypes V1016G/F1534C	ALIVE	DEAD
SS/RS	**1**	**1**
SS/RR	**22**	**20**
RS/RS	**25**	**15**
RR/SS	**8**	**5**
chisquare test pvalue *0.56*		
